# A High-Performance and Interpretable p*K*_a_ Prediction Framework Integrating Count-Based Fingerprints and Ensemble Learning

**DOI:** 10.3390/molecules31060961

**Published:** 2026-03-12

**Authors:** Hui Shen, Yongquan He, Juefeng Deng, Xiaoying Li, Chenqiang Yang, Dingren Ma, Dehua Xia, Haiying Yu

**Affiliations:** 1 Zhejiang Key Laboratory of Digital Intelligence Monitoring and Restoration of Watershed Environment, College of Geography and Environmental Sciences, Zhejiang Normal University, Jinhua 321004, China; shenhui182@163.com (H.S.); 13694832946@163.com (X.L.); hbkjdxyang@163.com (C.Y.); 2 China Energy Conservation and Environmental Protection Group, Techand Environmental Protection Group Co., Ltd., Guangzhou 510330, China; heyongquan@sztechand.com.cn (Y.H.); dengjuefeng@sztechand.com.cn (J.D.); 3 Guangdong Provincial Key Laboratory of Environmental Pollution Control and Remediation Technology, School of Environmental Science and Engineering, Sun Yat-Sen University, Guangzhou 510275, China; xiadehua3@mail.sysu.edu.cn

**Keywords:** p*K*_a_ prediction, count-based Morgan fingerprint, machine learning, quantitative structure-property relationship, applicability domain

## Abstract

The acid dissociation constant (p*K*_a_) is a fundamental parameter governing the environmental fate of organic compounds. Accurate p*K*_a_ prediction remains challenging, as traditional binary Morgan fingerprints (B-MF) fail to capture stoichiometric information critical for modeling substituent effects. This study developed an interpretable machine learning framework for p*K*_a_ prediction by integrating count-based Morgan fingerprints (C-MF) with ensemble algorithms. Through systematic comparison across four algorithms (Catboost, XGBoost, GBDT, RF), C-MF consistently outperformed B-MF due to its ability to quantify functional group multiplicity. Subsequent SHAP-based recursive feature elimination (SHAP-RFE) optimized the model, identifying Catboost with only 81 features as the optimal architecture, achieving a test-set *R*^2^ of 0.890 and *RMSE* of 1.026. SHAP analysis revealed that the model’s decisions are driven by chemically intuitive features, forming a hierarchical framework where primary ionizable sites set the baseline p*K*_a_ and electronic modifiers fine-tune it. The applicability domain, defined using the AD_SAL_ method, yielded high-confidence predictions (*R*^2^ = 0.926). External validation on an independent open-source dataset containing 6876 acidic compounds, combined with results from AD_SAL_ application domain characterization, enabled accurate p*K*_a_ prediction for 390 compounds within the application domain (*R*^2^ = 0.890, *RMSE* = 0.942). This further confirms the model’s strong generalizability. This work provides a robust and generalizable tool for high-performance p*K*_a_ prediction, with significant potential for applications in environmental risk assessment.

## 1. Introduction

The acid dissociation constant (p*K*_a_) is a fundamental physicochemical parameter that governs the ionization equilibrium of organic compounds in aqueous solution. As a quantitative measure of acid–base strength, p*K*_a_ critically influences the chemical behavior and environmental fate of organic molecules [1,2,3]. Accurate p*K*_a_ prediction is therefore essential for evaluating key processes such as membrane permeability, metabolic transformation, environmental bioavailability, and ecotoxicity, constituting an indispensable component of early-stage environmental risk assessment [4,5].

Classic p*K*_a_ prediction methods primarily rely on linear quantitative structure-property relationship (QSPR) frameworks. Techniques based on multiple linear regression utilize molecular descriptors such as structural descriptors and quantum chemical descriptors to construct predictive models. These methods laid the foundation for p*K*_a_ prediction development, with their core advantages being clear chemical mechanisms, high interpretability, and low computational cost. Alcázar et al. [6] combined low-cost quantum mechanical calculations with QSAR methodology, and achieved accurate prediction of basic p*K*_a_ of a wide range of nitrogen-containing compounds, with an *R*^2^ of 0.9905, outperforming commercial Chemaxon software (version 25.1.X). However, the inherent limitations of linear QSPR methods have become increasingly apparent. They fail to cover the vast chemical space and struggle to capture nonlinear synergistic electronic effects among multiple substituents, thereby restricting their application in predicting the p*K*_a_ values of structurally diverse novel organic pollutants in environmental contexts.

The rapid development of machine learning (ML) has driven the widespread application of diverse algorithms, including ensemble learning, artificial neural networks, support vector machines, and deep learning, in accurate, high throughput p*K*_a_ predictive models [7,8,9,10,11,12]. These approaches effectively compensate for the limitations of linear models and demonstrate outstanding predictive performance across structurally diverse organic compounds. Representative studies have confirmed the advantages of nonlinear machine learning in p*K*_a_ prediction. Fazeli et al. [10] found that the feedforward neural network (FFNN) model outperformed linear GA-MLR and particle swarm optimization-support vector machine (PSO-SVM) in predicting amino acids, achieving a test set *R*^2^ of 0.9987; Rafieezade et al. [8] further confirmed that nonlinear PSO-SVM achieved optimal performance in predicting p*K*_a_ values of amino groups in amino acids, with an *R*^2^ of 0.9681, significantly outperforming linear models.

In molecular characterization methods, researchers employ various molecular descriptors, such as structure descriptors, empirical descriptors, and quantum chemical descriptors, to characterize the structural information of compounds [9,13]. Additionally, Morgan fingerprints (also known as extended connectivity fingerprints) have become a widely used feature in chem-informatics-driven p*K*_a_ prediction due to their ability to encode the local atomic environment in a rotationally invariant manner [14,15]. Yang et al. [7] developed a high-performance p*K*_a_ prediction model in response to the core requirements of drug discovery, using a technical route that combines machine learning (graph neural networks (GNNs); deep learning methods) with molecular fingerprints. Their acidic and basic models achieved mean absolute errors (*MAE*) of 0.621 and 0.402 on the test set, respectively, demonstrating excellent predictive performance and validating the effectiveness of the Morgan fingerprint method in p*K*_a_ prediction tasks [7]. However, Traditional binary Morgan fingerprints are typically implemented in binary form, merely recording the presence or absence of specific substructures. While effective for many classification tasks, this binary representation inherently discards stoichiometric information—it cannot distinguish whether a functional group appears once or multiple times in a molecule [16,17]. For p*K*_a_ prediction, where acid–base strength is directly influenced by the number and proximity of ionizable groups, this limitation becomes critical, constraining model accuracy for multifunctional compounds.

The count-based Morgan fingerprints (C-MF) address this limitation by quantifying the occurrence frequency of each substructure within a molecule [18]. This enriched representation provides a more granular characterization of molecular composition, potentially capturing nuances that are critical for predicting properties, which are influenced by the multiplicity of functional groups and local electronic environments. In environmental science, C-MF has been applied to tackle relevant challenges. For instance, Zhong et al. [17] employed C-MF to characterize pollutant structures and constructed machine learning models for activity and property prediction. Results demonstrated that across 10 datasets, C-MF outperformed B-MF in 9 datasets, revealing the potential advantage of count-based fingerprints in predicting physicochemical properties of compounds [17]. However, the application of C-MF specifically for predicting compound p*K*_a_ values remains unexplored, and no study has systematically verified its performance advantage and mechanism in p*K*_a_ prediction.

In summary, to address this research gap, this study systematically developed and validated a high-performance, interpretable p*K*_a_ prediction framework by integrating C-MF with ensemble ML. We first rigorously compared the predictive performance of models built on C-MF and B-MF across multiple algorithms. The optimal model architecture was then identified through SHAP-based recursive feature elimination (SHAP-RFE). The interpretability of the final model was elucidated by decoding the high-contribution features back to key chemical substructures. Furthermore, the model’s applicability domain (AD) was rigorously defined, and its strong generalizability was confirmed via external validation. This work improves the generalization performance of p*K*_a_ prediction across a broader chemical space through enhanced fingerprint encoding and feature-selection strategies, providing a reliable and transparent computational tool for p*K*_a_ estimation, with direct utility in environmental fate and risk assessment.

## 2. Results and Discussion

### 2.1. Comparative Performance Assessment of B-MF and C-MF

**Figure 1:**
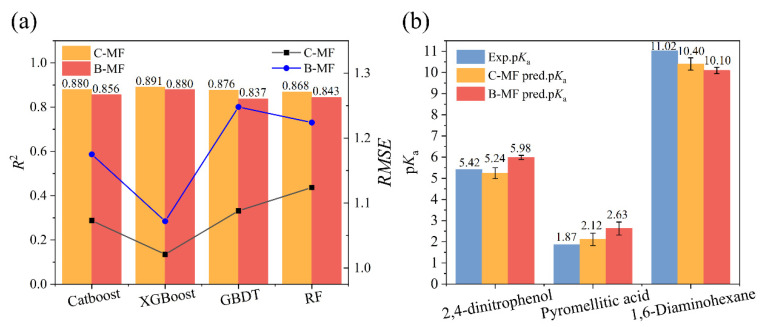
(**a**) Performance comparison (*R*^2^ and *RMSE*) between B-MF and C-MF across ML algorithms; (**b**) Predicted results for B-MF and C-MF in three representative compounds.

The encoding logic of molecular fingerprints fundamentally influences the information available to ML models. B-MF only denotes the presence or absence of specific substructures, whereas C-MF quantifies their frequency. To systematically evaluate the impact of this representational difference on p*K*_a_ prediction accuracy, models based on B-MF and C-MF were rigorously compared using an identical dataset. Four ensemble ML algorithms—Catboost, XGBoost, GBDT, and RF—were employed to ensure the robustness of the comparison. Model performance was assessed using *R*^2^ and *RMSE* on a held-out test set. The comparative performance of the two fingerprint representations is visualized in Figure 1a, with comprehensive metrics detailed in Table S3.

Across all four algorithms (Figure 1a), models utilizing C-MF consistently and significantly outperformed their B-MF counterparts, demonstrating superior predictive accuracy and stability, as evidenced by higher *R*^2^ and lower *RMSE* values. Moreover, the advantage of C-MF was particularly evident with algorithms capable of high-precision fitting. For instance, the XGBoost model with C-MF achieved an *R*^2^ of 0.891 and an *RMSE* of 1.021, surpassing the B-MF-based model (*R*^2^ = 0.880, *RMSE* = 1.072). Even for the RF algorithm, which generally exhibits weaker generalization for complex chemical mixtures, the adoption of C-MF led to a clear improvement, increasing *R*^2^ by 0.016 and reducing *RMSE* by 0.064.

Although the theoretical superiority of C-MF over B-MF is supported by the well-established substituent electronic effect additivity principle, this systematic comparison is still of critical scientific necessity for three core reasons. First, there is a clear disconnect between theoretical chemical principles and practical application in the current p*K*_a_ prediction field: most existing ML-based p*K*_a_ prediction models still widely use B-MF as the core molecular representation, and no study has systematically verified the application value of C-MF in p*K*_a_ prediction [6,8,10]. Second, this study quantifies the performance improvement boundary and chemical mechanism of C-MF for p*K*_a_ prediction, confirming that C-MF reduces the mean absolute error of multi-functional compounds by 50% through representative cases. Third, this comparison confirms that the performance improvement comes from the optimization of molecular representation rather than the algorithm itself, laying a core foundation for subsequent feature selection and mechanistic interpretability analysis. This result is also consistent with prior findings that C-MF has superior performance in the property prediction of environmental pollutants [17].

The consistent performance advantage of C-MF is attributed to its retention of stoichiometric information, which is essential for accurately predicting the p*K*_a_ of compounds containing multiple specific functional groups. For example, the presence of several electron-withdrawing or electron-donating functional groups is a key factor influencing p*K*_a_, as they modulate acid dissociation strength by altering electron density [19]. The predictions for three representative multifunctional compounds, summarized in Figure 1b, validate the rationality of this mechanism.

For 2,4-dinitrophenol (SMILES: O=[N+](c1c(O)ccc([N+]([O^−^])=O)c1)[O^−^], a classic compound featuring two strongly electron-withdrawing nitro groups (-NO_2_) and one ionizable phenolic hydroxyl group (-OH), C-MF explicitly quantifies the two -NO_2_ groups. This enables the model to accurately capture their combined electron-withdrawing effect, which stabilizes the conjugate base and lowers the p*K*_a_, resulting in a prediction (5.24) with an absolute error of only 0.18 (experimental value: 5.42). In contrast, B-MF merely notes the “presence” of a -NO_2_ group, failing to distinguish between one or two such groups, which leads to a less accurate prediction (5.99, error: 0.57).

The case of pyromellitic acid (SMILES: O=C(O)c(c(cc(c1C(=O)O)C(=O)O)C(=O)O)c1), which features four ionizable carboxyl groups (-COOH) attached to an aromatic ring, further demonstrates the advantage of the count-based representation. The enhanced acidity of this compound, relative to monocarboxylic acids, arises from the strong synergistic inductive and electrostatic interactions among the multiple -COOH groups. By quantifying the exact number of -COOH groups, the C-MF-based model accurately captures this effect, yielding a predicted p*K*_a_ of 2.12 (absolute error = 0.25) against an experimental value of 1.87. In contrast, the B-MF model, limited to binary presence/absence encoding, fails to resolve the substituent multiplicity, resulting in a less accurate prediction of 2.63 (absolute error = 0.76).

The linear diamine 1,6-diaminohexane (SMILES: NCCCCCCN) provides a key example of the model’s ability to handle multifunctional bases. The molecule contains two terminal primary amino groups (–NH_2_), both of which are protonatable and collectively elevate the basicity relative to a monoamine. The C-MF representation quantifies the presence of two such groups, providing the model with explicit information about the increased number of basic sites. Consequently, the C-MF-based ensemble models yielded a mean predicted p*K*_a_ of 10.40, corresponding to an absolute error of 0.80 relative to the experimental value (11.20). In contrast, the B-MF models, which only register the presence of an –NH_2_ group, produced a lower and less accurate mean prediction of 10.10, with a larger absolute error of 1.10.

Across these three representative cases, C-MF reduced the mean absolute error (*MAE*) by 50% (0.41 vs. 0.81 for B-MF), conclusively demonstrating its robustness as a molecular representation for p*K*_a_ prediction. This finding validates the critical value of stoichiometric information in capturing the effects of substituent multiplicity. It establishes a solid foundation for model optimization, particularly for complex multifunctional molecules where precise quantification of functional group interactions is paramount for accurate physicochemical property prediction.

### 2.2. Dimensionality Reduction and Optimal Model Framework Selection

Based on the comparative results above, C-MF was selected for further modeling. However, the high-dimensional and potentially redundant nature of its feature set can adversely affect model generalizability and interpretability. To address this, SHAP-RFE was applied to the C-MF descriptors. The feature selection process was guided by monitoring *R*^2^ on the validation set during iterative feature pruning, with the objective of identifying a minimal yet impactful subset of fingerprints. As shown in Table 1, SHAP-RFE successfully streamlined the C-MF descriptor set while simultaneously improving predictive accuracy across all algorithms, effectively balancing model performance with feature efficiency. Moreover, a comparative analysis of evaluation metrics (*R*^2^ and *RMSE*) and the size of the retained feature subsets revealed distinct trade-offs between predictive power and computational practicality of the four different algorithms.

**Figure 2:**
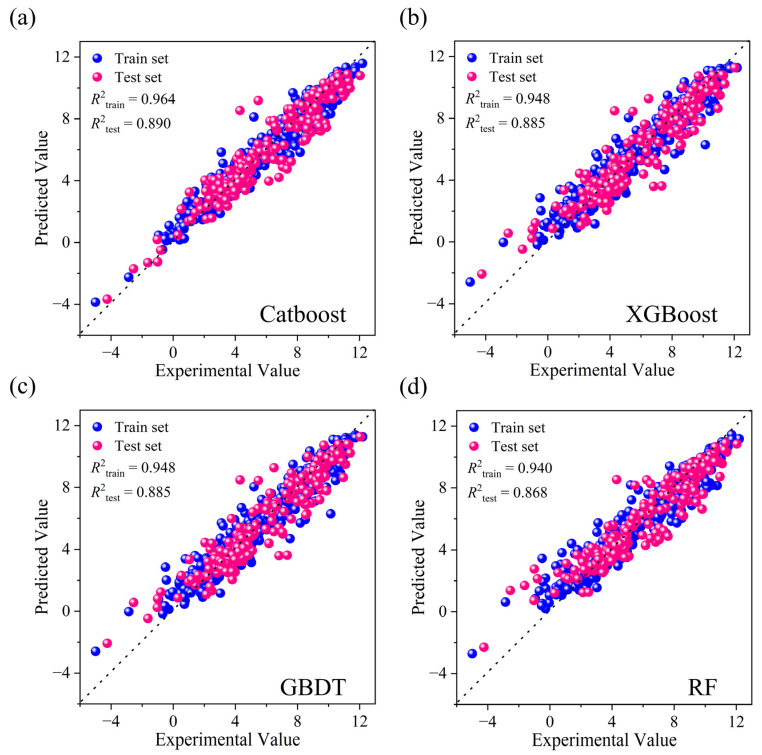
Comparison of experimental and predicted values for four models after SHAP-RFE feature selection: (**a**) Catboost; (**b**) XGBoost; (**c**) GBDT; (**d**) RF.

Specifically, Catboost achieved the most efficient feature representation, retaining the smallest subset of only 81 features (Text S3), while simultaneously delivering high predictive accuracy. Its test-set *R*^2^ increased from 0.880 to 0.890, with the *RMSE* decreasing from 1.073 to 1.026 (Figure 2). In contrast, although XGBoost attained marginally superior test-set metrics (*R*^2^ = 0.891, *RMSE* = 1.023), it required the largest feature subset (451 features)—over five times that of Catboost. This minimal performance gain (Δ*R*^2^ = 0.001, Δ*RMSE* = 0.003) is offset by the substantially higher model complexity, which increases computational cost and the risk of overfitting. Both GBDT (101 features) and RF (111 features) also demonstrated improved performance after SHAP-RFE, increasing test set *R*^2^ values to 0.885 and 0.868, respectively. However, their predictive accuracy remained lower than that of Catboost and XGBoost while utilizing 25–37% more features than the most parsimonious Catboost model.

In summary, SHAP-RFE effectively enhanced model performance while achieving significant dimensionality reduction. More importantly, the integration of SHAP-RFE-pruned C-MF with the Catboost algorithm established an optimal p*K*_a_ prediction framework, offering a superior balance among prediction accuracy, model efficiency, and generalizability.

### 2.3. Interpretability and Chemical Significance of SHAP-Derived Features

**Figure 3:**
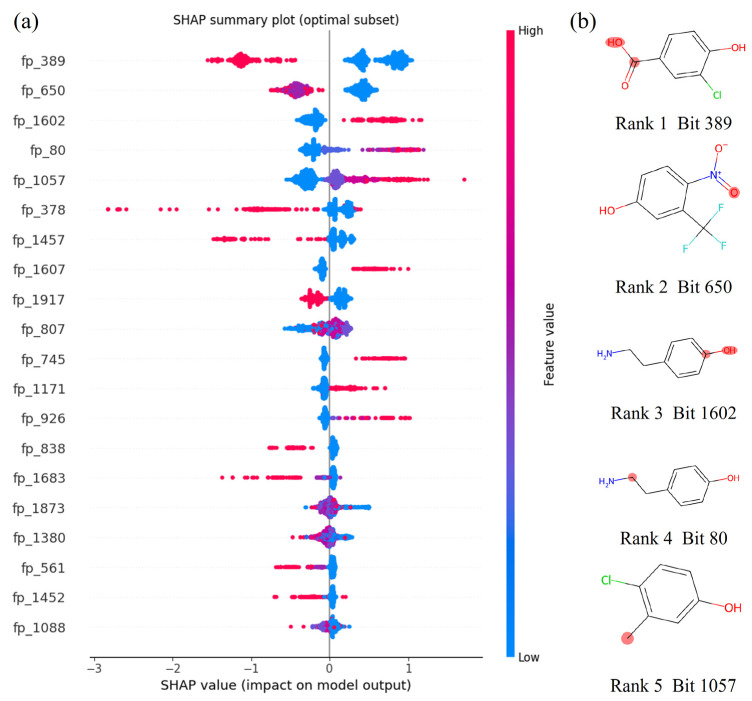
(**a**) SHAP feature contributions in the Catboost model; (**b**) Molecular structures corresponding to the top five fingerprint features ranked by SHAP contribution in the Catboost model.

To elucidate the chemical meaning behind the high-importance features identified by SHAP analysis, the corresponding fingerprint positions were decoded back to their original chemical substructures. This reverse mapping provides a direct link between the model’s learned weights and explicit chemical groups. As illustrated in Figure S1, the relative contribution of feature types varied across the four evaluated models, reflecting their distinct learning characteristics. The optimal Catboost model (Figure 3) was selected for in-depth mechanistic interpretation, enabling a clear attribution of SHAP contributions to specific functional subunits and clarification of their structural roles and chemical functions.

As shown in Figure 3, the top-ranked feature, corresponding to fingerprint bit 389 (SMARTS: #6=[#8]), was decoded as the carboxyl group scaffold, -C(=O)OH. This functional group is the primary acidic moiety in numerous organic molecules, and its high SHAP value directly signifies its crucial role in lowering p*K*_a_. The underlying mechanism involves the carbonyl oxygen (C=O) withdrawing electron density through resonance, which stabilizes the resulting carboxylate conjugate base (-COO^−^) and thus facilitates proton dissociation [20]. This established interplay of resonance and inductive effects is the fundamental reason for the characteristic low p*K*_a_ range (typically 1–5 for monocarboxylic acids) of carboxylic acids [20,21].

The model’s precision in recognizing this dominant acidic site is validated by subgroup analysis based on carboxyl group count. Predictions for compounds containing one carboxyl group (*N* = 370) yielded an *MAE* of 0.396, which is significantly lower than the *MAE* of 0.606 for compounds without any carboxyl groups (*N* = 743). Furthermore, the model accurately captures the enhanced acidity of dicarboxylic acids arising from the synergistic electron-withdrawing effect between adjacent carboxyl groups. For example, for a long-chain dicarboxylic acid (SMILES: O=C(O)CCCCCC(=O)O, experimental p*K*_a_ = 4.51), the predicted p*K*_a_ was 4.27 (*MAE* = 0.24), correctly reflecting a lower p*K*_a_ compared to monocarboxylic acids like acetic acid (p*K*_a_ ≈ 4.76) [22]. In essence, this high-importance feature acts as a primary detector for the most impactful acidic site, enabling the model to accurately prioritize key ionizable groups and effectively discern acidic molecules.

The second-most important feature, corresponding to fingerprint bit 650 (SMARTS: [#7+]-[#8-]), was decoded as the nitro group motif, N(=O)O^−^. This functional group is a prototypical strong electron-withdrawing group (EWG) that profoundly influences p*K*_a_ by depleting electron density from adjacent ionizable sites, such as the phenolic hydroxyl group. The underlying electronic effect involves both inductive and resonance components, which synergistically stabilize the resultant negatively charged conjugate base (e.g., a phenoxide anion) and thereby lower the p*K*_a_ [23].

The model’s predictive accuracy aligns closely with this established chemical mechanism. For example, 2,4-dinitrophenol (SMILES: c1(c(cccc1N(=O)=O)O)N(=O)=O, experimental p*K*_a_ = 4.96) was predicted with high accuracy (predicted p*K*_a_ = 5.09, *MAE* = 0.13). Across the entire dataset, dinitrophenols (*N* = 17) had a mean experimental p*K*_a_ of 3.79 and a mean predicted p*K*_a_ of 3.77, yielding a negligible mean deviation of 0.02. In contrast, mononitrophenols (*N* = 41) exhibited a significantly higher mean experimental p*K*_a_ of 5.31 (mean predicted: 5.14, *MAE* = 0.17). Critically, the model successfully captured the substantial experimental p*K*_a_ depression of approximately 1.52 units associated with the second nitro group, predicting a difference of 1.37 units—a discrepancy of only 0.15 units. This close agreement confirms that the model reliably quantifies the enhanced electron-withdrawing effect of multiple nitro substituents [24]. Unlike the B-MF that merely notes the presence of a nitro group, the SHAP weight associated with Feature 650 in the C-MF framework effectively encodes the cumulative electron-withdrawing strength by accounting for group count. This allows the model to resolve the significant experimental p*K*_a_ gap (~1.52 units) between mono- and dinitrated phenols, highlighting a key advantage for predicting the effect of substituent multiplicity [25].

The third-most important feature, corresponding to fingerprint bit 1602 (SMARTS: #6:[#6]), was identified as the phenolic hydroxyl group (-Ar-OH). This feature is critical for predicting p*K*_a_ in the distinct range typical of phenols (approximately 8–11), a property governed by the resonance stabilization of the resulting phenoxide anion (-Ar-O^−^). This mechanism differentiates phenols from aliphatic alcohols, which have much higher p*K*_a_ values (≈16–18) and are effectively non-ionizable under common environmental or physiological conditions [26].

The model’s performance validates the precise attribution of this feature. For monophenolic compounds—the largest subgroup in this category—predictions were highly accurate, with the lowest mean absolute error (*MAE* = 0.42) among all phenol-containing molecules. For instance, the p*K*_a_ of acetylphenol (experimental: 8.05) was accurately predicted as 8.28 (*MAE* = 0.23), demonstrating the model’s ability to correctly account for the moderate electron-withdrawing effect of the acetyl substituent, which lowers the p*K*_a_ relative to unsubstituted phenol (p*K*_a_ ≈ 9.99). The prediction error increased only slightly for compounds with two or more phenolic hydroxyl groups (*MAE* ≈ 0.48), indicating that the model robustly handles the added complexity, such as potential intramolecular hydrogen bonding, while maintaining core predictive accuracy. Functionally, this descriptor acts as a selective filter, enabling the model to distinguish the uniquely ionizable phenolic moiety from aliphatic alcohols and to accurately target its characteristic intermediate p*K*_a_ range.

Collectively, the three highest-weighted SHAP-derived features construct a hierarchical and chemically interpretable framework for p*K*_a_ prediction. Primary ionizable groups, specifically carboxyl and phenolic hydroxyl moieties, establish the baseline p*K*_a_ range intrinsic to the molecule. Electronic modifiers such as the nitro group fine-tune this acidic tendency by modulating electron density at the ionization site. The model’s ability to accurately capture these direct (ionization) and indirect (electronic modulation) physicochemical mechanisms confirms the mechanistic robustness of the Catboost model. More importantly, this high degree of interpretability not only validates the model’s predictive performance but also underpins its generalizability, as the identified features correspond to fundamental chemical drivers of acidity that are broadly applicable across organic compounds.

### 2.4. Definition and Evaluation of Applicability Domain

The AD of the optimal Catboost model, constructed using the C-MF, was characterized using the AD_SAL_ method. The final AD was defined by the parameters: *ρ*_s,T_ ≥ 1.000 and *I*_A,T_ ≤ 1.025. The stringent parameter selection reflects the intrinsic characteristics of the dataset: a high *ρ*_s,T_ threshold ensures that only compounds with adequate structural similarity to the training set are predicted, while a low *I*_A,T_ limit mitigates the influence of potential “activity cliffs” caused by minor structural variations that significantly alter p*K*_a_. This configuration prioritizes high prediction reliability, making the model particularly suitable for accuracy-critical tasks such as guiding experimental validation.

**Figure 4:**
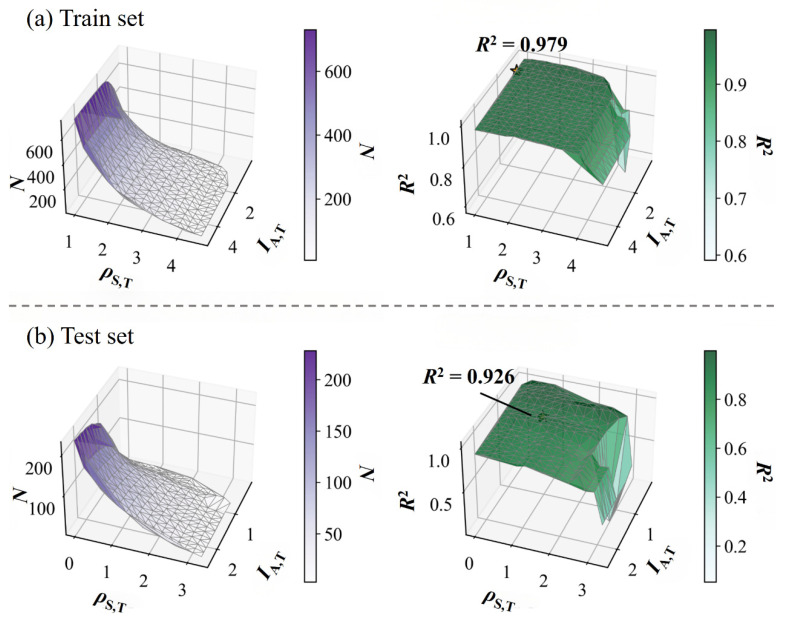
Catboost model performance (*R*^2^) versus the number of compounds retained in the AD (*N*) for the (**a**) source domain (Train set) and (**b**) target domain (Test set) (The yellow stars in the Figure 4 indicate the relative positions of the optimal applicability domain parameter combinations (*ρ*_s,T_ and *I*_A,T_) on both the training and test sets, as well as their corresponding *R*^2^ values).

Under this defined domain, 62 of the 228 compounds in the independent test set (27.19%, Table S4 and Figure 4) were included. For these in-domain compounds, the model achieved excellent predictive accuracy (*R*^2^ = 0.926, Figure 4). Notably, the model demonstrated strong overall generalization capability. Even without restricting predictions to the AD, its performance on the entire test set remained robust (*R*^2^ = 0.891, *RMSE* = 1.026, Table S4). The established AD, therefore, provides a practical, standardized guideline for identifying high-confidence predictions, offering a valuable tool for reliable p*K*_a_ assessment in environmental fate modeling, toxicity evaluation, and other scenarios where predictive certainty is paramount.

### 2.5. External Dataset Validation

**Figure 5:**
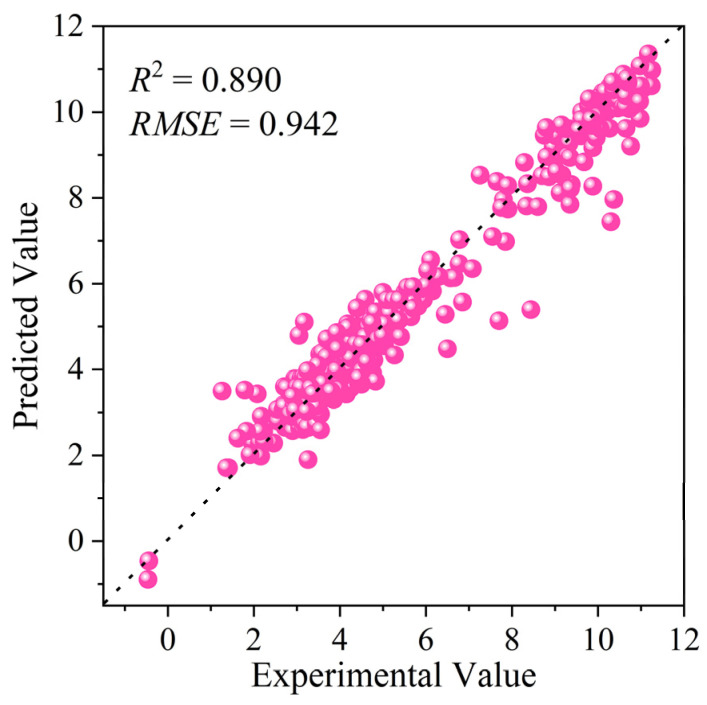
Comparison of experimental versus predicted p*K*_a_ values for compounds in the open-source dataset that meet the AD_SAL_ criteria {*ρ*_s,T_ ≥ 1.000, *I*_A,T_ ≤ 1.025}.

To rigorously assess the generalization capability of the optimized Catboost model and the appropriateness of its defined AD, an external validation was performed using an independent, open-source p*K*_a_ dataset (sourced from DataWarrior) [27,28]. The initial dataset contained 7912 compounds. To maintain a clear focus on acidic behavior, amphoteric compounds were excluded, resulting in a final validation set of 6876 distinct acidic compounds. Applying the pre-defined AD_SAL_ criteria (*ρ*_s,T_ ≥ 1.000, *I*_A,T_ ≤ 1.025), 390 compounds were identified as within the model’s AD (Table S5). For these in-domain compounds, the model demonstrated excellent predictive performance, achieving an *R*^2^ of 0.890 and an *RMSE* of 0.942 against the experimental values (Figure 5). These results confirm the model’s strong generalizability and the practical utility of the established AD for reliable p*K*_a_ estimation on novel, external compounds.

### 2.6. Performance Comparison with State-of-the-Art pK_a_ Prediction Models

To clarify the advantages of the interpretable and high-performance p*K*_a_ prediction model proposed in this study compared to those in previous research, we systematically conducted qualitative and quantitative comparisons between our optimal model and representative machine learning p*K*_a_ prediction frameworks reported in recent literature.

From a qualitative perspective, the core characteristics of our model are compared with two representative frameworks in Table 2. Compared with the deep learning-based model of Yang et al. [7] and the multi-algorithm open-source model of Mansouri et al. [12], our model has the following advantages: (1) it uses pure SMILES-driven C-MF representation without relying on quantum chemical calculations, achieving ultra-high computational efficiency suitable for high-throughput screening; (2) Through SHAP-RFE feature selection, reducing C-MF to 81 key features achieved outstanding predictive performance, enhancing the model’s generalization capability while reducing its complexity; (3) it has complete chemical interpretability through SHAP-based feature decoding, rather than a “black-box” prediction; and (4) it strictly defines the AD of the model, providing a clear guideline for high-confidence prediction.

## 3. Materials and Methods

### 3.1. Dataset Construction

The dataset was constructed by integrating experimentally determined p*K*_a_ values from published literature. It comprises 580 organic oxygen-containing acids and 563 organic nitrogen-containing bases, yielding a total of 1143 unique compounds that span 15 distinct structural classes [29,30,31,32,33,34,35,36,37,38,39]. The sample distribution and p*K*_a_ range for each compound class are detailed in Table S1.

### 3.2. Generation of Molecular Fingerprint

Molecular fingerprints were generated from the Simplified Molecular-Input Line-Entry System (SMILES) representation of each compound. For this purpose, the RDKit cheminformatics toolkit was employed within a Python environment. Specifically, binary Morgan fingerprints (B-MFs) were computed using the “AllChem.GetMorganFingerprintAsBitVect” function, which encodes the presence or absence of radial substructures. In contrast, C-MFs were generated using the “AllChem.GetHashedMorganFingerprint” function, which retains the occurrence frequency of each substructural pattern, thereby incorporating stoichiometric information.

The fundamental distinction between the two fingerprint representations lies in their feature encoding logic. B-MF generates a bit vector where each position is assigned a value of 0 or 1, indicating only the presence or absence of a specific substructural fragment within the molecule. In contrast, C-MF produces an integer vector where each position holds a non-negative integer, directly quantifying the frequency of occurrence of each fragment. This difference is chemically consequential. For example, for the substructure corresponding to a nitro group, B-MF would assign an identical value of 1 to both 4-nitrophenol (one nitro group) and 2,4-dinitrophenol (two nitro groups), failing to capture the difference in substituent count. C-MF, however, would encode this feature as 1 and 2, respectively, thereby preserving the stoichiometric information essential for modeling quantitative electronic effects.

To enhance model performance, the key parameters of the Morgan fingerprints—namely, the search radius and the bit vector length (nbits)—were systematically optimized. A Bayesian optimization approach was employed to efficiently explore the hyperparameter space and identify the configuration yielding the best predictive accuracy for our dataset. This process resulted in the selection of a radius of 1 and a bit length of 2048 for both B-MF and C-MF representations.

### 3.3. Model Development and Evaluation

A systematic modeling workflow was implemented to ensure robust training and reliable performance evaluation. This workflow consisted of four key stages: (1) dataset partitioning, (2) algorithm selection and implementation, (3) hyperparameter optimization, and (4) comprehensive performance assessment using established metrics. All computational procedures were performed within a Python 3.12 environment using Jupyter Notebook (version 7.3.2) to ensure reproducibility.

#### 3.3.1. Dataset Partitioning

To ensure a rigorous and unbiased evaluation, the dataset was partitioned into training, validation, and independent test sets using a stratified splitting strategy. This method preserved the proportional distribution of all compound subclasses across each subset, preventing the overrepresentation of dominant classes and enhancing the generalizability of the evaluation. Specifically, the data were first split in an 80:20 ratio to create a combined training set and a held-out test set. Subsequently, 20% of the training set was further allocated as a validation set. This resulted in a final distribution of 64% for training, 16% for validation, and 20% for independent testing. The validation set was used to monitor overfitting and guide hyperparameter tuning during model development, while the completely unseen test set provided the final assessment of model performance.

#### 3.3.2. Ensemble Learning Algorithms

Four state-of-the-art ensemble learning algorithms were employed to develop robust p*K*_a_ prediction models, selected for their proven efficacy in modeling complex, nonlinear structure–property relationships and handling high-dimensional molecular descriptors [40]. The selected algorithms were: (1) Random Forest (RF): An ensemble method that constructs multiple decision trees via bagging and random feature selection, effectively mitigating overfitting and providing stable predictions [41]; (2) Gradient Boosting Decision Tree (GBDT): A boosting technique that builds decision trees sequentially, with each new tree learning to correct the residuals of the previous ensemble, thereby optimizing for predictive accuracy; (3) Extreme Gradient Boosting (XGBoost): An optimized and scalable implementation of gradient boosting that incorporates L1 and L2 regularization for enhanced overfitting control and computational efficiency; (4) Catboost: A gradient boosting algorithm specifically designed to handle categorical features efficiently and reduce overfitting through ordered boosting. Its robustness with high-dimensional data makes it particularly suitable for modeling complex molecular fingerprints [42]. These algorithms collectively provide a comprehensive framework for comparing fingerprint representations and identifying the most effective model architecture for p*K*_a_ prediction.

#### 3.3.3. Hyperparameter Optimization

The hyperparameters for each ensemble algorithm were optimized using Bayesian optimization, a probabilistic global optimization method that efficiently explores the parameter space by building a surrogate model of the objective function and leveraging prior evaluation results. This approach typically converges faster and achieves better performance than conventional grid or random search [43]. The optimization was implemented using the Optuna framework. For each algorithm, critical hyperparameters were tuned within predefined search ranges (detailed in Table S2).

#### 3.3.4. Model Performance Evaluation

Model performance was systematically evaluated using two standard regression metrics: the coefficient of determination (*R*^2^) and the root mean square error (*RMSE*) [40,44]. *R*^2^ quantifies the proportion of variance in p*K*_a_ values explained by the model, with values closer to 1.0 indicating a better fit. *RMSE* measures the average magnitude of the prediction errors, where lower values correspond to higher precision. Performance was assessed on the training, validation, and independent test sets. Furthermore, to ensure the model’s fairness and generalizability across diverse chemistries, its predictive accuracy was also evaluated within each major compound subclass, thereby identifying and mitigating any potential structural bias.

### 3.4. SHAP-Based Recursive Feature Elimination

To enhance model interpretability, reduce the dimensionality of the high-dimensional fingerprint vectors, and identify a minimal yet predictive feature subset, a SHAP-based recursive feature elimination (SHAP-RFE) strategy was implemented. SHAP (SHapley Additive exPlanations) provides a theoretically grounded framework for quantifying the contribution of each feature to model predictions, offering a more rigorous alternative to conventional feature-importance metrics in tree-based models [45]. Previous studies have demonstrated that combining molecular fingerprinting with SHAP-based interpretability effectively enhances the mechanism-based interpretability of QSPR models [9]. The SHAP-RFE procedure consists of three sequential steps: (1) SHAP-based feature ranking: The trained model using the full fingerprint set is explained using the training data, and features are ranked in descending order according to their mean absolute SHAP value; (2) Recursive elimination: Starting from the full set, the least important feature (lowest SHAP rank) is iteratively removed. After each removal, the model is retrained and evaluated on a held-out validation set. The feature subset that maximizes the *R*^2^ on the validation set is selected as optimal; (3) Final model construction: The prediction model is rebuilt using only the optimal feature subset, thereby reducing complexity while preserving—or even improving—predictive accuracy.

### 3.5. Model Mechanism Interpretation

To elucidate the chemical meaning behind the high-importance features identified by SHAP analysis (Figure S1), the corresponding fingerprint positions were decoded back to their original molecular substructures. This reverse mapping and subsequent structural visualization were conducted using the RDKit cheminformatics toolkit, a widely adopted open-source resource for molecular informatics (Text S1).

### 3.6. Defining the Applicability Domain

The AD of the optimal p*K*_a_ prediction model was characterized using the AD_SAL_ method, a structure-activity landscape-based approach, to delineate the chemical space within which reliable predictions can be expected (Text S2) [46]. The AD is defined by two parameters: the molecular similarity density threshold (*ρ*_s,T_) and the activity inconsistency threshold (*I*_A,T_). A query compound is considered within the AD if it satisfies the dual criteria *ρ*_s,q_ ≥ *ρ*_s,T_ and *I*_A,q_ ≤ *I*_A,T_, ensuring that it resides within a well-sampled region of the training chemical space and is not subject to local “activity cliffs”. This defined AD was applied to evaluate prediction confidence on both the internal test set and an external validation set sourced from DataWarrior [27,28]. The final list of compounds within the AD and their corresponding predictions is provided in the Supporting Information.

## 4. Conclusions

In this study, a robust and interpretable ML framework for p*K*_a_ prediction was developed by integrating C-MF with ensemble algorithms, specifically to address the limitations of B-MF in capturing stoichiometric information. Systematic comparisons demonstrated the consistent superiority of C-MF across multiple algorithms (Catboost, XGBoost, GBDT, RF) as it quantitatively encodes the frequency of key substructures—a capability critical for accurately modeling the electronic effects of multiple functional groups. Subsequent optimization via SHAP-RFE feature selection yielded a parsimonious yet high-performance model, with Catboost utilizing a minimal set of 81 features identified as the optimal framework. Mechanistic interpretation of the top SHAP-derived features—carboxyl groups, nitro groups, and phenolic hydroxyl groups—revealed a chemically intuitive hierarchy: primary ionizable sites establish the baseline p*K*_a_, while electron-regulating groups fine-tune acidity. Furthermore, the AD was rigorously defined using the AD_SAL_ method, which enabled high-confidence predictions. The model’s strong external validation performance confirms its excellent generalizability. Collectively, this work delivers an efficient, interpretable, and generalizable tool for high-performance p*K*_a_ prediction, with significant value for environmental science applications—including chemical risk assessment, fate modeling, and the design of green chemicals—where accurate estimation of acid-base behavior is essential. Follow-up research will further integrate electronic structure properties such as electrostatic potential with the C-MF framework to further improve the predictive accuracy and mechanistic interpretability of the model for a wider range of organic compounds.

## Figures and Tables

**Table 1:** Performance of C-MF-based p*K*_a_ prediction models after SHAP-RFE.

ML Algorithm	SHAP-RFE	Train Set		Validation Set		Test Set		Feature Number
		*R* ^2^	*RMSE*	*R* ^2^	*RMSE*	*R* ^2^	*RMSE*	
Catboost	Before	0.955	0.645	0.823	1.180	0.880	1.073	2048
	After	0.964	0.580	0.833	1.144	0.890	1.026	81
XGBoost	Before	0.959	0.616	0.841	1.117	0.891	1.030	2048
	After	0.961	0.603	0.843	1.110	0.891	1.023	451
GBDT	Before	0.924	0.839	0.838	1.128	0.876	1.088	2048
	After	0.948	0.697	0.836	1.135	0.885	1.051	101
RF	Before	0.933	0.790	0.820	1.187	0.859	1.160	2048
	After	0.940	0.749	0.823	1.179	0.868	1.124	111

**Table 2:** Qualitative comparison with representative p*K*_a_ prediction models.

Model	Core Molecular Representation	Optimal Algorithm	Full Chemical Interpretability	Strict AD Definition	High Computational Cost Required
This study	C-MF (SMILES-driven)	Catboost	Yes (SHAP-based substructure decoding and hierarchical mechanism analysis)	Yes (ADSAL dual-parameter method)	No
Yang et al. [7]	Graph-based molecular descriptors	Graph neural network	Partial (attention weight visualization)	No systematic definition	Yes
Mansouri et al. [12]	Multi-type 2D molecular descriptors	RF/SVM	Limited (only overall feature importance ranking)	No systematic definition	No

## Data Availability

The raw data and processed datasets supporting the findings of this study are openly available in the figshare repository at https://doi.org/10.6084/m9.figshare.31474897. 1. External validation dataset in domain.csv; 2. External validation dataset out domain.csv; 3. External validation dataset.csv; 4. training dataset.csv.

## Supplementary Materials

The following supporting information can be downloaded at: https://www.mdpi.com/article/10.3390/molecules31060961/s1, including compounds included in the model dataset (Table S1), optimal hyperparameters for each model after dimension reduction (Table S2), the specific models constructed using B-MF and C-MF (Table S3), domain parameters of the Catboost (Table S4), experimental and predicted values of p*K*_a_ for compounds within the external validation domain and their application domain parameters (Table S5), SHAP maps of the top 20 features with the highest SHAP contributions for each model after dimensionality reduction (Figure S1), using RDKit to identify the structure corresponding to the molecular fingerprint (Text S1), details on AD_SAL_ {*ρ*_s,q_ ≥ *ρ*_s,T_, *I*_A,q_ ≤ *I*_A,T_} characterization (Text S2), the optimal molecular fingerprint feature indices obtained for the Catboost model after SHAP-RFE (Text S3).

## Abbreviations

The following abbreviations are used in this manuscript:

AD	Multidisciplinary Digital Publishing Institute
ADSAL	Directory of open access journals
B-MF	Three letter acronym
C-MF	Linear dichroism
EWG	Electron-withdrawing group
GBDT	Gradient Boosting Decision Tree
*MAE*	Mean absolute error
ML	Machine learning
QSPR	Quantitative structure-property relationship
p*K*_a_	Acid dissociation constant
*R*^2^	Coefficient of determination
RF	Random Forest
*RMSE*	Root mean square error
SHAP-RFE	SHAP-based recursive feature elimination
XGBoost	Extreme Gradient Boosting
