# Supplementary material for: A High-Performance and Interpretable pKa Prediction Framework Integrating Count-Based Fingerprints and Ensemble Learning
Source: Molecules. 2026 Mar 12;31(6):961. doi: 10.3390/molecules31060961 (PMC13029067; doi:10.3390/molecules31060961)
Supplement: Supplementary file 1 [file molecules-31-00961-s001.zip › molecules-4168159-supplementary.pdf]

Supplementary Information

for

A High-Performance and Interpretable pKa Prediction Framework Integrating Count-Based Fingerprints and Ensemble Learning

Hui Shen [1], Yongquan He [2], Juefeng Deng [2], Xiaoying Li [1], Chenqiang Yang [1], Dingren Ma [1,*], Dehua Xia [3] and Haiying Yu [1,*]

1 Zhejiang Key Laboratory of Digital Intelligence Monitoring and Restoration of Watershed Environment, College of Geography and Environmental Sciences, Zhejiang Normal University, Jinhua 321004, China; shenhui182@163.com (H.S.); 13694832946@163.com (X.L.); hbkjdxyang@163.com (C.Y.)

2 China Energy Conservation and Environmental Protection Group, Techand Environmental Protection Group Co., Ltd., Guangzhou 510330, China; heyongquan@sztechand.com.cn (Y.H.); dengjuefeng@sztechand.com.cn (J.D.)

3 Guangdong Provincial Key Laboratory of Environmental Pollution Control and Remediation Technology, School of Environmental Science and Engineering, Sun Yat-Sen University, Guangzhou 510275, China; xiadehua3@mail.sysu.edu.cn

* Correspondence: madingren@zjnu.edu.cn (D.M.); yhy@zjnu.cn (H.Y.).

Content

Table S1. Compounds included in the model dataset.. 3

Table S2. Optimal hyperparameters for each model after dimension reduction......... 28

Table S3. The specific models constructed using B-MF and C-MF respectively....... 29

Table S4. Domain parameters of the Catboost.. 30

Table S5. Experimental and predicted values of pK_a for compounds within the external validation domain and their application domain parameters 31

Figure S1. SHAP maps of the top 20 features with the highest SHAP contributions for each model after dimensionality reduction: (a) Catboost; (b) XGBoost; (c) GBDT; (d) RF 49

Text S1. Use RDKit to identify the structure corresponding to the molecular fingerprint.. 50

Text S2. Details on ADSAL $\{\rho s,q \geq \rho s,T, IA,q \leq IA,T\}$ characterization 51

Test S3. The optimal molecular fingerprint feature indices obtained for the Catboost model after SHAP-RFE... 52

Table S1. Compounds included in the model dataset

Num	SMILES	Exp.pK_a	Type
1	NCCc1ccc(O)cc1	9.77	1
2	Cc1c(Cl)ccc(O)c1	9.2	1
3	CC(Cc1ccc(O)cc1)(C)C	10.43	1
4	c1(cc(O)ccc1[N+](=O)[O-])C(F)(F)F	6.07	1
5	c1(Cl)c(C)cc(cc1C)O	9.7	1
6	c1(ccc(cc1)O)c2ccccc2	9.55	1
7	C(OCCCC)(=O)c1ccc(cc1)O	8.47	1
8	c1(Cl)c(Cl)ccc(O)c1	8.63	1
9	c1(C)cc(O)ccc1C	10.36	1
10	C(C)(C)(C)c1ccc(cc1)O	10.39	1
11	C(F)(F)(F)c1cccc(O)c1	8.95	1
12	C(C)(=O)c1ccc(cc1)O	8.05	1
13	c1(C(C)C)ccc(cc1)O	10.24	1
14	O=Cc1cccc(O)c1	8.98	1
15	[N+](=O)([O-])c1ccc(cc1)O	7.15	1
16	C(C)(=O)Nc1ccc(cc1)O	9.38	1
17	c1(ccc(cc1)O)Cl	9.41	1
18	c1(O)ccc(cc1)C	10.26	1
19	c1(ccc(cc1)O)Br	9.17	1
20	c1(ccccc1)O	9.99	1
21	c1(O)cc(C)cc(C)c1	10.19	1
22	c1c(cccc1Cl)O	9.12	1
23	c1c(C)cccc1O	10.09	1
24	C(OCC)(=O)c1ccc(cc1)O	8.34	1
25	c1(cccc(O)c1)C(C)=O	9.25	1
26	c1(ccc(cc1)O)N	10.45	1
27	O=Cc1ccc(cc1)O	7.61	1
28	c1(O)ccc(cc1)CC	10	1
29	c1(O)ccc(cc1)OC	10.1	1
30	c1c(cccc1O)OC	9.65	1
31	c1(ccc(cc1)O)F	9.91	1
32	c1c(cccc1F)O	9.21	1
33	C(F)(F)(F)c1ccc(cc1)O	8.68	1
34	c1(O)cc(OC)cc(OC)c1	9.34	1
35	c1(ccc(cc1)O)I	9.21	1
36	[N+](=O)([O-])c1cccc(O)c1	8.36	1
37	c1(cc(O)ccc1[N+](=O)[O-])[N+](=O)[O-]	5.42	1
38	c1(cccc(O)c1)c2ccccc2	9.64	1
39	c1(cccc(O)c1)C(C)(C)C	10.12	1
40	[N+](=O)([O-])c1cc(O)cc(c1)[N+](=O)[O-]	6.69	1
41	c1(Cl)cc(O)cc(Cl)c1	8.18	1
42	c1c(cccc1N)O	9.86	1
43	c1c(cccc1Br)O	9.03	1
44	c1(Cl)c(Cl)cc(cc1Cl)O	7.84	1
45	c1(cccc(O)c1)C(C)C	10.16	1

46	c1c(CC)cccc1O	9.9	1
47	c1c(cccc1O)OCC	9.65	1
48	c1(O)ccc(cc1)OCC	10.13	1
49	c1(Br)cc(O)cc(Br)c1	8.06	1
50	c1c(cccc1I)O	9.03	1
51	c1(O)ccc(cc1)CCC	10.34	1
52	c1(O)cc(CC)cc(C)c1	10.1	1
53	N#Cc1ccc(cc1)O	7.97	1
54	N#Cc1cccc(O)c1	8.61	1
55	c1(O)ccc(cc1)CS	9.53	1
56	c12CCCc1ccc(O)c2	10.32	1
57	[N+](=O)([O-])c1c(C)ccc(O)c1	8.62	1
58	C(C)(=O)c1ccc(cc1)O	8.05	1
59	O=C(OC(c1cccc2)(c(ccc(O)c3)c3)c(ccc(O)c4)c4)c12	9.7	1
60	c1cc(O)ccc1C(c2ccc(O)cc2)c3ccccc3CO	9.65	1
61	Oc(cccc1O)c1	9.32	1
62	Oc(cc(O)cc1O)c1	8.45	1
63	Oc(ccc(O)c1)c1	10.85	1
64	Oc(ccc(c1ccc2)c2)c1	9.51	1
65	OCc1cccc(O)c1	9.83	1
66	OCc1ccc(O)cc1	9.82	1
67	Oc1cc2CCCCc2cc1	10.48	1
68	Oc1ccc(cc1)CCCCCCCCC	10.25	1
69	Oc1ccc(cc1)C(C)CC(C)CC(C)C	11.06	1
70	Oc(cc(C)c1N(=O)=O)cc1C	8.25	1
71	S(=O)(=O)(C)c(ccc1)cc1O	9.33	1
72	Ic(cc(I)c1)cc1O	8.1	1
73	Oc(cc(c1)OCC)cc1OCC	9.37	1
74	O=Nc(ccc(O)c1)c1	6.48	1
75	O=S(=O)(c1ccc(O)cc1)C	7.83	1
76	O=S(=O)(c1c(cc(O)cc1C)C)C	8.13	1
77	Oc1cc(cc(c1)C(C)(C)C)C(C)(C)C	10.29	1
78	c1(ccccc1O)C(N)=O	6.49	1
79	Oc(cc(c(c1C)C)C)c1	10.25	1
80	c1(ccccc1O)C(N)=O	8.89	2
81	C(=O)(Nc1ccccc1)c2ccccc2O	7.4	2
82	c1(ccccc1O)C(OC)=O	9.87	2
83	c1(O)c(cccc1C=O)OC	7.91	2
84	c1(cc(cc(Cl)c1O)Cl)C(=O)Nc2ccc(cc2)Cl	4.7	2
85	C(=O)(Nc1ccccc1Cl)c2ccccc2O	7.31	2
86	c1(c(O)ccc(c1)N(=O)=O)C(=O)Nc2ccccc2	3.03	2
87	C(=O)(Nc1ccc(cc1)Br)c2ccccc2O	7.31	2
88	c1(cc(ccc1O)Br)C(=O)Nc2ccc(cc2)Cl	6	2
89	C(=O)(Nc1ccc(cc1)Cl)c2ccccc2O	7.3	2
90	c1(cc(cc(Cl)c1O)Cl)C(=O)Nc2ccccc2	4.7	2
91	c1(cc(ccc1O)Cl)C(=O)Nc2ccccc2	6.17	2
92	c1(cc(ccc1O)Cl)C(=O)Nc2ccccc2C	6.6	2
93	C(=O)(Nc1ccc(Cl)cc1Cl)c2ccccc2O	7.14	2

94	N(=O)(=O)c1ccccc1NC(=O)c2ccccc2O	6.91	2
95	c1(cc(ccc1NC(=O)c2ccccc2O)Cl)N(=O)=O	6.74	2
96	c1(ccc(Br)c(C)c1O)C(NC)=O	7.52	2
97	c1(cc(cc(Cl)c1O)Cl)C(=O)Nc2ccc(cc2)F	4.8	2
98	c1(cc(cc(Br)c1O)Br)C(=O)Nc2ccc(Cl)cc2N(=O)=O	4.11	2
99	C(=O)(Nc1ccc(Cl)cc1C)c2ccccc2O	7.43	2
100	c1(cc(ccc1O)F)C(=O)Nc2ccc(Br)cc2C	7.1	2
101	c1(cc(ccc1O)F)C(=O)Nc2ccc(Cl)cc2C	7.3	2
102	c1(cc(cc(Br)c1O)Br)C(=O)Nc2ccc(F)cc2F	4.77	2
103	c1(cc(cc(Cl)c1O)Cl)C(=O)Nc2ccc(F)cc2F	4.77	2
104	c1(cc(cc(Cl)c1O)Cl)C(=O)Nc2ccc(Cl)cc2N(=O)=O	4.11	2
105	c1(cc(cc(Cl)c1O)Cl)C(=O)Nc2ccc(cc2C)N(=O)=O	4.41	2
106	O=Cc(c(O)ccc1)c1	8.34	2
107	O=C(c(c(O)ccc1)c1)C	9.19	2
108	Clc1ccc2oc(nc2c1)c1cc(N)ccc1O	9.81	2
109	c1(c(O)ccc(c1)[N+](=O)[O-])[N+](=O)[O-]	4.09	3
110	c1(Cl)c(O)c(Cl)cc(Cl)c1Cl	5.22	3
111	c1(c(cccc1N(=O)=O)O)N(=O)=O	4.96	3
112	c1(Cl)c(Cl)c(O)c(c(Cl)c1Cl)Cl	4.7	3
113	c1(O)c(cccc1Cl)Cl	6.79	3
114	c1(O)c(C)cccc1Cl	8.69	3
115	c1(cc(cc([N+](=O)[O-])c1O)[N+](=O)[O])[N+](=O)[O-]	0.38	3
116	c1(cc(Cl)cc([N+](=O)[O-])c1O)[N+](=O)[O-]	2.96	3
117	c1(cc(cc(C(C)CC)c1O)[N+](=O)[O-])[N+](=O)[O-]	4.62	3
118	[N+](=O)([O-])c1ccccc1O	7.23	3
119	c1(ccccc1O)C(C)C	10.47	3
120	c1(ccccc1O)C(C)(C)C	10.28	3
121	c1(O)c(Cl)cc(cc1Cl)Cl	6.23	3
122	c1(O)cc(C)ccc1C(C)C	10.62	3
123	c1(C(C)C)c(O)cc(c(Cl)c1)C	9.98	3
124	[N+](=O)([O-])c1c(O)ccc(Cl)c1	6.46	3
125	c1(ccccc1O)c2ccccc2	9.92	3
126	c1(ccccc1OC)O	9.98	3
127	c1(ccccc1CC)O	10.2	3
128	c1(OC)cc(C)ccc1O	10.28	3
129	c1(ccccc1OCC)O	10.11	3
130	c1c(Cl)c(O)cc(Cl)c1Cl	7.4	3
131	c1(O)cc(C)ccc1C	10.41	3
132	c1(ccccc1O)Cl	8.56	3
133	c1(ccccc1O)Br	8.45	3
134	c1(ccccc1O)N	9.75	3
135	c1(ccccc1C)O	10.28	3
136	c1(c(O)ccc(C(C)(C)C)c1)C(C)(C)C	11.72	3
137	c1(OC)cc(ccc1O)C=CC	9.88	3
138	c1(OC)cc(CC=C)ccc1O	10.19	3
139	c1(ccc(c(Cl)c1)O)C(C)(C)C	8.58	3
140	c1(ccc(c(C)c1)O)C(C)(C)C	10.59	3

141	[N+](=O)([O-])c1ccc(c(N)c1)O	7.6	3
142	[N+](=O)([O-])c1cc(Br)c(c(Br)c1)O	3.39	3
143	c1(C)cc(C)ccc1O	10.6	3
144	c1(O)c(Br)cc(cc1Br)Br	6.8	3
145	N(=O)(=O)c1c(O)ccc(N)c1	7.81	3
146	[N+](=O)([O-])c1cc(C)ccc1O	7.4	3
147	c1(Cl)c(O)ccc(Cl)c1	7.89	3
148	c1(OC)cc(ccc1O)C=O	7.4	3
149	c1(O)c(cccc1C(C)(C)C)C(C)(C)C	11.7	3
150	c1(O)c(cc(cc1C(C)(C)C)C)C(C)(C)C	12.23	3
151	c2(cc(cc(C1CCCCC1)c2O)[N+](=O)[O-])[N+](=O)[O-]	4.52	3
152	[N+](=O)([O-])c1ccc(cc1O)[N+](=O)[O-]	5.21	3
153	c1(ccccc1O)F	8.7	3
154	[N+](=O)([O-])c1ccc(cc1O)F	6.07	3
155	c1(O)c(C)cc(c(C)c1)C	10.57	3
156	c1(C)c(C)cccc1O	10.54	3
157	c1(O)c(C)cc(cc1C)C	10.86	3
158	c1(ccccc1O)I	8.51	3
159	c1(c(O)c(C)cc(c1)[N+](=O)[O-])[N+](=O)[O-]	4.31	3
160	c1(O)c(O)ccc(CCN)c1C	9.54	3
161	[N+](=O)([O-])c1cccc(c1O)[N+](=O)[O-]	3.97	3
162	c1(O)c(C)cccc1C	10.62	3
163	c1(Cl)c(cccc1Cl)O	7.7	3
164	c1(O)c(Cl)ccc(Cl)c1	7.51	3
165	[N+](=O)([O-])c1cccc(Cl)c1O	5.48	3
166	c1(Br)c(Br)c(O)c(c(Br)c1Br)Br	4.62	3
167	c1(O)c(cccc1Br)Br	6.67	3
168	c1(cc(C)cc([N+](=O)[O-])c1O)[N+](=O)[O-]	4.23	3
169	N#Cc1ccccc1O	6.86	3
170	[N+](=O)([O-])c1ccc(cc1O)Cl	6.05	3
171	c1(Br)c(O)ccc(Br)c1	7.79	3
172	[N+](=O)([O-])c1cc(Cl)c(c(Cl)c1)O	3.55	3
173	[N+](=O)([O-])c1ccc(c(Cl)c1)O	5.45	3
174	c1(O)cc(ccc1OC)C=O	8.89	3
175	c1(ccccc1CCC)O	10.47	3
176	c1(O)cc(C)cc(C)c1C	10.67	3
177	c1(C)cc(c([N+]([O-])=O)cc1)O	7.41	3
178	c2(cc(cc(c1ccccc1)c2O)[N+](=O)[O-])[N+](=O)[O-]	3.85	3
179	c1(C(C)(C)C)cc(C(C)(C)C)cc(C(C)(C)C)c1O	12.19	3
180	c1(F)c(F)c(O)c(c(F)c1F)F	5.53	3
181	[N+](=O)([O-])c2c(O)ccc(c1ccccc1)c2	6.73	3
182	c1(Cl)c(Cl)ccc(Cl)c1O	5.8	3
183	c1(O)c(Cl)c(Cl)cc(Cl)c1Cl	5.14	3
184	c1(cc(cc(Cl)c1O)[N+](=O)[O-])[N+](=O)[O-]	2.1	3
185	[N+](=O)([O-])c1c(O)ccc(OC)c1	7.31	3
186	c1(C)cc(Cl)ccc1O	9.71	3
187	c1(O)c(Br)cc(cc1Br)C#N	3.86	3

188	c1(cc(C)cc(C)c1O)C(C)(C)C	12.04	3
189	c1(O)c(cccc1C(C)C)C(C)C	11.1	3
190	c1(cc(C)ccc1O)C(C)(C)C	11.72	3
191	[N+](=O)([O-])c1cc(C)c(c(C)c1)O	7.07	3
192	c1(O)c(Cl)cc(cc1Cl)C	7.19	3
193	c1(O)c(Cl)cc(cc1Cl)Br	6.21	3
194	[N+](=O)([O-])c1c(O)ccc(C(C)CC)c1	7.59	3
195	C(O)(=O)c1ccc(O)c(Cl)c1	7.52	3
196	c1(Cl)c(Cl)c(O)cc(Cl)c1Cl	6.35	3
197	N(=O)(=O)c1ccc(c(O)c1)C	8.59	3
198	Cl-c(cc(c1)C)c(c1)O	8.74	3
199	c2(c(ON=Cc1cc(Br)c(c(Br)c1)O)ccc(c2)N(=O)=O)N(=O)=O	5.46	3
200	Oc(c(c(c(c1)Cl)Cl)Cc(c(c(cc2Cl)Cl)Cl)c2O)c1Cl	4.95	3
201	OCc(c(O)ccc1)c1	9.84	3
202	Oc(c(c(ccc1)cc2)c1)c2	9.34	3
203	Oc(c(cc(c1)Cl)Cc(c(O)ccc2Cl)c2)c1	7.6	3
204	Oc(c(O)ccc1)c1	9.45	3
205	c1c(O)c2CCCCc2cc1	10.28	3
206	Oc(c(c(c(O)c1)ccc2)c2)c1	9.58	3
207	Oc(c(c(c(c1)Cl)ccc2)c2)c1	8.86	3
208	Oc1ccccc1C	10.22	3
209	Oc1c(I)cc(C#N)cc1I	3.96	3
210	Oc1ccccc1CCCC	10.58	3
211	c1c(O)c(CO)cc(C)c1	10.15	3
212	Oc(c(c(c(c1)C)C)C)c1	10.59	3
213	Oc(c(c(O)cc1)C)c1	10.05	3
214	Oc1cccc2c1CCC2	10.32	3
215	Oc(c(ccc1)CC=C)c1	10.28	3
216	Oc(c(cc(c1)Cl)Cc(c(O)c(cc2Cl)Cl)c2)c1Cl	5.6	3
217	Oc1ccc(Cl)c(Cl)c1Cl	6.5	3
218	Oc1cccc(O)c1O	9.01	3
219	Oc1cc(Cl)c(O)c(Cl)c1	7.38	3
220	N#Cc1cc(C)c(O)c(C)c1	8.27	3
221	[O-][N+](=O)c1cc(c(O)c(c1)C(C)(C)C)C(C)(C)C	6.62	3
222	Brc1ccc(O)c(Cl)c1	7.64	3
223	Oc1ccc(cc1Cl)c1ccccc1	8.07	3
224	Oc(c(cc1CCC)CCC)c(c1)CCC	11.47	3
225	C(O)(=O)c1ccc(c(Cl)c1)Cl	3.64	4
226	c1(cc(Cl)cc(Cl)c1)C(O)=O	3.54	4
227	S(=O)(=O)(N(CCC)CCC)c1ccc(cc1)C(O)=O	3.4	4
228	[N+](=O)([O-])c1ccc(cc1)C(O)=O	3.44	4
229	C(O)(c1ccccc1)=O	4.19	4
230	C(O)(=O)c1ccc(cc1)Cl	3.98	4
231	C(O)(=O)c2ccc1c(cccc1)c2	4.17	4
232	C(O)(=O)c1ccc(c(OC)c1)OC	4.36	4
233	C(O)(=O)c1ccc(cc1)C(C)(C)C	4.4	4
234	C(O)(=O)c1ccc(cc1)O	4.54	4

235	C(O)(=O)c1ccc(cc1)C	4.37	4
236	C(O)(=O)c1ccc(c(O)c1)O	4.48	4
237	[N+](=O)([O-])c1cc(cc(c1)[N+](=O)[O-])C(O)=O	2.82	4
238	c1(cc(O)cc(O)c1)C(O)=O	4.04	4
239	C(O)(=O)c1cccc(N)c1	4.74	4
240	C(O)(=O)c1cccc(C)c1	4.27	4
241	C(O)(=O)c1ccc(cc1)OC	4.47	4
242	[N+](=O)([O-])c1cccc(c1)C(O)=O	3.46	4
243	C(O)(=O)c1ccc(c(OC)c1)O	4.51	4
244	c1(O)c(O)cc(cc1O)C(O)=O	4.21	4
245	C(O)(=O)c1ccc(cc1)N	4.85	4
246	C(O)(=O)c1cccc(F)c1	3.86	4
247	C(O)(=O)c1ccc(cc1)F	4.14	4
248	c1(cc(ccc1[N+](=O)[O-])C(O)=O)[N+](=O)[O-]	2.82	4
249	c1(O)c(OC)cc(cc1OC)C(O)=O	4.34	4
250	C(O)(=O)c1cccc(Cl)c1	3.81	4
251	C(O)(=O)c1ccc(cc1)C(C)C	4.35	4
252	C(O)(=O)c1cccc(Br)c1	3.81	4
253	C(O)(=O)c1ccc(cc1)C(C)=O	3.7	4
254	C(O)(=O)c1ccc(cc1)Br	4	4
255	C(O)(=O)c1cccc(OC)c1	4.09	4
256	C(O)(=O)c1cccc(I)c1	3.85	4
257	C(O)(=O)c1ccc(cc1)OCC	4.45	4
258	c(cc(c1)C(=O)O)c(c1)C=O	3.77	4
259	N#Cc1ccc(cc1)C(O)=O	3.55	4
260	C(O)(=O)c1ccc(cc1)CC	4.35	4
261	C(O)(=O)c1ccc(cc1)I	4	4
262	C(O)(=O)c1cccc(c1)C=O	3.84	4
263	c1(cc(OC)cc(OC)c1)C(O)=O	3.97	4
264	C(O)(=O)c1cccc(c1)C#N	3.6	4
265	C(O)(=O)c1ccc(cc1)Oc2ccccc2	4.52	4
266	C(O)(=O)c2cccc(Oc1ccccc1)c2	3.92	4
267	S(C)(=O)(=O)c1ccc(cc1)C(O)=O	3.64	4
268	C(O)(=O)c1ccc(cc1)OCCC	4.46	4
269	O=C(O)c(cccc1O)c1	4.3	4
270	O=C(O)c(ccc(c1)C(=O)O)c1	3.51	4
271	O=C(O)c(cccc1C(=O)O)c1	3.7	4
272	OC(C1=CC(F)=CC(F)=C1)=O	3.59	4
273	OC(=O)c1cc(c(cc1)F)F	3.83	4
274	O=C(O)c(cc(cc1C(=O)O)C(=O)O)c1	3.12	4
275	OC(=O)c1cc(c(c(c1)F)F)F	3.54	4
276	c(cc(c1)S(=O)(=O)C)cc1C(=O)O	3.52	4
277	OC(=O)c1ccc(CN)cc1	3.59	4
278	OC(=O)c1ccc(S(=O)(=O)N)cc1	3.47	4
279	O=C(O)c(cc(cc1C)C)c1	4.3	4
280	O=C(Nc(ccc(c1)C(=O)O)c1)C	4.28	4
281	OC(=O)c1cc(NC(=O)C)ccc1	4.07	4
282	O=C(O)c(ccc(c1C)C)c1	4.41	4

283	O=C(Oc(ccc(c1)C(=O)O)c1)C	4.38	4
284	O=C(O)c1cccc(c1)C(C)(C)C	4.2	4
285	O=C(O)c(ccc(OC)c1N(=O)=O)c1	3.72	4
286	O=C(O)c(ccc(c1N(=O)=O)C)c1	3.62	4
287	O=C(O)c(ccc(c1N(=O)=O)Cl)c1	3.29	4
288	OC(=O)c1cccc(c1)C(F)(F)F	3.75	4
289	OC(=O)c1ccc(N(C)C)cc1	5.03	4
290	O=C(O)c(ccc(N(=O)=O)c1C)c1	3.65	4
291	COc1ccc(cc1C)C(O)=O	4.35	4
292	O=C(O)c1cc(OC)c(cc1)C	4.13	4
293	OC(=O)c1cc(Br)c(C)cc1	3.29	4
294	Cc1cc(ccc1Cl)C(O)=O	4.07	4
295	c1(ccc(cc1O)C)C(O)=O	3.4	5
296	c1(ccc(cc1Cl)Cl)C(O)=O	2.68	5
297	c1(cc(Cl)ccc1Cl)C(O)=O	2.47	5
298	c1(ccccc1OC(C)=O)C(O)=O	3.49	5
299	c1(c(Cl)ccc(Cl)c1Cl)C(O)=O	1.5	5
300	c1(c(cccc1Cl)Cl)C(O)=O	1.59	5
301	c1(ccc(cc1OCC)N)C(O)=O	5.09	5
302	c1(ccccc1Nc2cccc(C)c2C)C(O)=O	4.2	5
303	c1(ccc(cc1O)N)C(O)=O	3.66	5
304	c1(ccccc1O)C(O)=O	2.97	5
305	c1(cccc(C)c1O)C(O)=O	2.95	5
306	c1(ccccc1I)C(O)=O	2.93	5
307	c1(ccccc1Br)C(O)=O	2.88	5
308	c1(ccc(cc1O)O)C(O)=O	3.11	5
309	c1(cc(C)ccc1O)C(O)=O	3.15	5
310	O=C(O)c(c(O)ccc1Br)c1	2.66	5
311	c1(ccccc1NC(C)=O)C(O)=O	3.4	5
312	c1(ccc(cc1OC)OC)C(O)=O	4.36	5
313	c1(ccccc1Nc2ccccc2)C(O)=O	3.99	5
314	c2(C(O)=O)c(O)cc1ccccc1c2	2.79	5
315	[N+](=O)([O-])c1ccc(c(C(O)=O)c1)O	2.12	5
316	[N+](=O)([O-])c1ccc(c(Cl)c1)C(O)=O	2.14	5
317	c1(ccccc1N)C(O)=O	4.95	5
318	c1(ccccc1Cl)C(O)=O	2.89	5
319	C(O)(=O)c1ccccc1C	3.98	5
320	c1(cc(I)ccc1O)C(O)=O	2.62	5
321	c1(C(O)=O)c(cc(cc1[N+](=O)[O-])[N+](=O)[O-])[N+](=O)[O-]	0.65	5
322	c1(cc(I)cc(I)c1O)C(O)=O	2.3	5
323	c1(cc(Cl)cc(N)c1Cl)C(O)=O	3.4	5
324	c1(cccc(O)c1O)C(O)=O	2.91	5
325	c1(c(cccc1O)O)C(O)=O	1.05	5
326	c1(cc(Cl)ccc1O)C(O)=O	2.65	5
327	c1(ccccc1F)C(O)=O	3.27	5
328	c1(cc(O)ccc1O)C(O)=O	2.95	5
329	[N+](=O)([O-])c1ccccc1C(O)=O	2.17	5

330	c1(ccccc1C(C)=O)C(O)=O	4.13	5
331	c1(ccccc1OC)C(O)=O	3.9	5
332	c1(cc(cc(C(O)=O)c1O)N(=O)=O)N(=O)=O	0.7	5
333	c1(cc(ccc1C(O)=O)[N+](=O)[O-])[N+](=O)[O-]	1.42	5
334	C(O)(=O)c1c(C)cccc1C	3.35	5
335	c1(C(O)=O)c(F)c(F)c(c(F)c1F)C	2	5
336	c1(ccccc1c2ccccc2)C(O)=O	3.46	5
337	c1(c(cccc1OC)OC)C(O)=O	3.44	5
338	c1(cccc(OC)c1OC)C(O)=O	3.98	5
339	c1(c(Cl)ccc(Cl)c1OC)C(O)=O	1.97	5
340	c1(ccccc1Oc2ccccc2)C(O)=O	3.53	5
341	c1(ccccc1C(C)C)C(O)=O	3.63	5
342	[N+](=O)([O-])c1ccc(c(C(O)=O)c1)Cl	2.17	5
343	[N+](=O)([O-])c1cccc(C(O)=O)c1Cl	2.02	5
344	c2c(NC(c1c(C(O)=O)cccc1)=O)cccc2	2.5	5
345	N(=O)(=O)c1cccc(Cl)c1C(O)=O	1.34	5
346	c1(c(C)cccc1Cl)C(O)=O	2.75	5
347	c1(ccccc1NCC(N)=O)C(O)=O	4.2	5
348	O=C(O)c(c(c(ccc1)cc2)c1)c2	3.6	5
349	O=C(O)c(c(ccc1)C(=O)O)c1	2.76	5
350	O=C(O)c(c(cc(c1C(=O)O)C(=O)O)C(=O)O)c1	1.87	5
351	OC(=O)c1c(F)cccc1F	2.85	5
352	OC(C1=C(C=C(C(F)=C1)F)F)=O	3.28	5
353	O=C(O)c(c(F)c(F)c(F)c1F)c1F	2.72	5
354	OC(=O)c1c(c(cc(c1F)F)F)F	2.71	5
355	O=C(O)c(c(c(ccc1)cc2cccc3)c1)c23	3.65	5
356	OC(=O)c1c(c(c(c(c1)F)F)F)F	3.08	5
357	OC(=O)c1c(F)cc(F)cc1	3.58	5
358	OC(=O)c1c(c(ccc1F)F)F	2.82	5
359	OC(=O)c1c(F)ccc(F)c1	3.3	5
360	OC(=O)c1c(c(ccc1)F)F	3.29	5
361	OC(=O)c1c(c(c(cc1)F)F)F	3.3	5
362	c(cc(N(=O)=O)c1N(=O)=O)cc1C(=O)O	1.85	5
363	Brc(ccc1)c(c1N(=O)=O)C(=O)O	1.37	5
364	c(cc(O)c1C(=O)O)cc1N(=O)=O	2.24	5
365	O=C(O)c(c(O)cc(O)c1)c1O	1.68	5
366	OC(=O)c1c(O)c(N(=O)=O)ccc1	1.87	5
367	O=C(O)c(c(ccc1)C(=O)c(cccc2)c2)c1	3.54	5
368	O=C(O)c(c(cc(c1)C)C)c1C	3.45	5
369	c(cc(O)c1C(=O)O)cc1C	3.32	5
370	c(cc(N(=O)=O)c1C(=O)O)cc1N(=O)=O	1.14	5
371	Cc1cccc(C(O)=O)c1C	3.77	5
372	O=C(O)c(c(N(=O)(=O))ccc1N(=O)(=O))c1	1.62	5
373	O=C(O)c(c(ccc1C)C)c1	3.99	5
374	O=C(O)c(c(cc(c1)C)C)c1	4.22	5
375	CCC1=CC=CC=C1C(=O)O	3.79	5
376	O=C(c1c(cc(cc1Br)Br)Br)O	1.41	5
377	c12c(C(=O)O)c(C)ccc1cccc2	3.11	5

378	OC(=O)c1c(C)c(C)cc(C)c1C	3.42	5
379	N(=O)(=O)c1c(C(=O)O)c(C)ccc1	1.87	5
380	Clc(ccc1Cl)c(c1C(=O)O)C(=O)O	1.46	5
381	O=C(O)c(c(c(N(=O)=O)cc1N(=O)=O)C)c1	2.97	5
382	c(cc(C)c1C(=O)O)cc1OC	3.46	5
383	O=Cc(c(ccc1)C(=O)O)c1	4.55	5
384	OC(=O)c1c(O)ccc(F)c1	2.7	5
385	C(O)(C(C)O)=O	3.86	6
386	n3(C(=O)c1ccc(cc1)Cl)c2ccc(cc2c(CC(O)=O)c3C)OC	4.5	6
387	C(=O)(c1ccc(cc1)N)NCC(O)=O	3.8	6
388	N13C(C(O)=O)C(SC1C(NC(c2ccccc2)=O)C3=O)(C)C	2.74	6
389	N13C(C(O)=O)C(SC1C(NC(=O)c2c(cccc2OC)OC)C3=O)(C)C	2.77	6
390	OC(CI)=O	3.15	6
391	C(C)(O)=O	4.76	6
392	C(CS)(O)=O	3.55	6
393	C(C)(Cl)(Cl)C(O)=O	1.79	6
394	C(O)(=O)C(C)(C)C	5.03	6
395	C(O)(c1ccccc1)(c2ccccc2)C(O)=O	3.05	6
396	C(F)(F)(F)C(O)=O	0.52	6
397	C(Cl)(Cl)(Cl)C(O)=O	0.51	6
398	C125C=CC(C(C(=O)O1)(C)C2C(C34CC(C(C3)(O)CCC45)=C)C(O)=O)O	4	6
399	C(O)(=O)C(Cl)Cl	1.26	6
400	C(O)(C(C)C)=O	4.84	6
401	C(CO)(O)=O	3.83	6
402	C(CCl)(O)=O	2.87	6
403	C(CC)(O)=O	4.88	6
404	C(CBr)(O)=O	2.89	6
405	C14(C)C(C(C)CCC(O)=O)CCC1C3C(C2(C)CCC(CC2CC3O)O)CC4O	4.98	6
406	c1(c(Cl)ccc(Cl)c1Cl)CC(O)=O	3.7	6
407	c1(cccc2ccccc12)CC(O)=O	4.23	6
408	c2(CC(O)=O)c1ccccc1[nH]c2	4.75	6
409	N13C(C(O)=O)C(SC1C(NC(COc2ccccc2)=O)C3=O)(C)C	2.79	6
410	C(O)(C(CC)CC)=O	4.71	6
411	C(O)(=O)C(O)c1ccccc1	3.41	6
412	c1c(Cl)c(Cl)cc(Cl)c1OCC(O)=O	2.83	6
413	c1(c(Cl)cc(c(Cl)c1)Cl)OC(C)C(O)=O	2.84	6
414	c1(Cl)c(OCCCC(O)=O)ccc(Cl)c1	4.95	6
415	c1(Cl)c(ccc(Cl)c1)OCC(O)=O	2.73	6
416	c1(C)cc(Cl)ccc1OCC(O)=O	3.13	6
417	C(O)(C(C)CCC)=O	4.79	6
418	C(O)(C1CCCCC1)=O	4.9	6
419	C(O)(C(CCC)CCC)=O	4.6	6
420	c1(O)cc(ccc1O)CC(O)=O	4.25	6

421	C(O)(Cc1ccccc1)=O	4.31	6
422	N(=O)(=O)c1ccc(cc1)CC(O)=O	3.85	6
423	C(O)(Cc1ccc(cc1)OC)=O	4.36	6
424	C(O)(=O)CCCl	3.99	6
425	C(CCC)(O)=O	4.82	6
426	C(O)(C(C)CC)=O	4.81	6
427	C(O)(=O)C(c1ccccc1)c2ccccc2	3.94	6
428	O(C(C)C(O)=O)c1ccc(cc1Cl)Cl	3.1	6
429	C(O)(COc1ccc(cc1)Cl)=O	3.1	6
430	C(O)(COc1ccccc1)=O	3.17	6
431	C(O)(CCC(C)=O)=O	4.64	6
432	C(C(O)=O)C(O)=O	2.85	6
433	C(O)(=O)CCI	4.09	6
434	C(CF)(O)=O	2.59	6
435	CC(CC(O)=O)O	4.41	6
436	C(CCCc1ccc(cc1)N(CCCl)CCCl)(O)=O	5.75	6
437	c1(OC)cc(ccc1O)CC(O)=O	4.41	6
438	C2(O)(C(O)=O)CC(OC(=O)C=Cc1ccc(O)c(O)c1)C(O<br>)C(O)C2	2.66	6
439	C(O)(Cc1cccc(F)c1)=O	4.13	6
440	C(O)(COc1ccccc1F)=O	3.08	6
441	N#CCC(O)=O	2.45	6
442	F-c(ccc1)cc1OCC(=O)O	3.13	6
443	C(O)(COc1ccc(cc1)F)=O	3.13	6
444	C(O)(Cc1ccc(cc1)F)=O	4.24	6
445	C(N)(=O)NCC(O)=O	3.89	6
446	OCC(C(O)=O)O	3.55	6
447	C(O)(CCc1ccccc1)=O	4.66	6
448	C(O)(CC(C)C)=O	4.77	6
449	OCCC(O)=O	4.51	6
450	C(C)(O)(c1ccccc1)C(O)=O	3.53	6
451	C(C)(C(O)=O)C(O)=O	3.12	6
452	OC(CCCCCC1SCC(=O)N1)=O	5.1	6
453	C(O)(Cc2ccc1c(cccc1)c2)=O	4.25	6
454	C(O)(COc1cccc(Cl)c1)=O	3.07	6
455	c1(Cl)c(Cl)ccc(OCC(O)=O)c1	2.92	6
456	C(O)(=O)C(C)(C)O	3.61	6
457	C(C)(C)(C(O)=O)C(O)=O	3.15	6
458	C(O)(=O)C(C)(C)CC	5.03	6
459	C(O)(C(C)Cl)=O	2.8	6
460	C(O)(C(C)Br)=O	2.97	6
461	C(CC)(C(O)=O)C(O)=O	2.96	6
462	C(O)(COc1ccccc1Cl)=O	3.05	6
463	C(CCC)(C(O)=O)C(O)=O	2.99	6
464	C(O)(C(CCC)O)=O	3.89	6
465	c(cc(c1)CC(=O)O)c(c1)C	4.37	6
466	C(O)(CCC(C)C)=O	4.84	6
467	OC(CN(C=O)O)=O	3.5	6

468	C(O)(COc1ccc(cc1)C)=O	3.21	6
469	C(O)(COc1cccc(C)c1)=O	3.2	6
470	C1(CC1)C(O)=O	4.83	6
471	C(O)(COc1cccc(Br)c1)=O	3.09	6
472	N(=O)(=O)c1ccc(cc1)OCC(O)=O	2.89	6
473	C(O)(Cc1ccc(cc1)I)=O	4.18	6
474	C(CCCc1ccccc1)(O)=O	4.76	6
475	C(O)(COc1ccc(cc1)OC)=O	3.21	6
476	N(=O)(=O)c1cccc(CC(O)=O)c1	3.97	6
477	C(O)(COc1ccc(cc1)I)=O	3.16	6
478	C(O)(COc1cccc(I)c1)=O	3.13	6
479	C(O)(COc1ccccc1I)=O	3.17	6
480	C(O)(COc1ccc(cc1)Br)=O	3.13	6
481	N(=O)(=O)c1cccc(OCC(O)=O)c1	2.95	6
482	[N+](=O)([O-])c1ccccc1OCC(O)=O	2.9	6
483	C(O)(COc1ccccc1OC)=O	3.23	6
484	N#Cc1ccc(cc1)OCC(O)=O	2.93	6
485	C(O)(Cc1cccc(I)c1)=O	4.16	6
486	C(O)(Cc1ccc(cc1)Br)=O	4.19	6
487	C(O)(Cc1ccc(cc1)Cl)=O	4.19	6
488	C(O)(Cc1cccc(Cl)c1)=O	4.14	6
489	C(O)(COc1ccccc1C)=O	3.23	6
490	N#Cc1cccc(c1)OCC(O)=O	3.03	6
491	C(O)(COc1ccccc1Br)=O	3.13	6
492	C(O)(COc1cccc(OC)c1)=O	3.14	6
493	C(CCCCc1ccccc1)(O)=O	4.88	6
494	C(O)(COc1cccc2ccccc12)=O	3.2	6
495	N2(CC(O)=O)c1c(cccc1SC2=O)Cl	3.04	6
496	C(O)(CC1CCCCC1)=O	4.8	6
497	C(O)(COc1ccccc1O)=O	3.02	6
498	C(O)(=O)C(O)c1ccc(cc1)OC	3.42	6
499	C(O)(Cc1ccc(cc1)CC)=O	4.37	6
500	N(c1ccccc1CC(O)=O)c2c(cccc2Cl)Cl	4.15	6
501	c(cc(c1)C(C(=O)O)C)c(c1)CC(C)C	4.45	6
502	OC(C(C)Oc1ccc(Cl)cc1C)=O	3.68	6
503	c(ccc1c(c2)OCCC(=O)O)cc1cc2	4	6
504	n2c(c1ccc(cc1)Cl)c(sc2c3ccccc3)CC(O)=O	3.6	6
505	C(O)(=O)Cc1ccccc1I	4.04	6
506	C(O)(C(CCC)CC)=O	4.71	6
507	C(c1ccccc1)(=O)c2cccc(C(C)C(O)=O)c2	4.45	6
508	c1(Cl)cc(ccc1OCC=C)CC(O)=O	4.29	6
509	c2(ccc1c(ccc(OC)c1)c2)C(C)C(O)=O	4.15	6
510	CC(c2cc(Oc1ccccc1)ccc2)C(O)=O	4.5	6
511	C(O)(Cc1ccc(cc1)C(C)(C)C)=O	4.42	6
512	C(CCC(O)=O)(=O)c1ccc(cc1)c2ccccc2	4.51	6
513	C3(=Cc1ccc(cc1)S(C)=O)c2ccc(cc2C(CC(O)=O)=C3<br>C)F	4.7	6
514	CC(c2ccc(C(c1sccc1)=O)cc2)C(O)=O	3.91	6

515	C(O)(=O)C(C)Oc1ccc(cc1)Oc2ccc(cc2Cl)Cl	3.43	6
516	OC(Cc3c(c1ccc(Cl)cc1)nn(c2ccccc2)c3)=O	4.3	6
517	n1c(Cl)c(Cl)cc(Cl)c1OCC(O)=O	2.68	6
518	C2(=O)c1ccccc1CSc3ccc(CC(O)=O)cc23	3.71	6
519	Cl-c(c(F)cc1)cc1N(C(=O)-c(ccc2)cc2)C(C(=O)O)C	3.72	6
520	n2c(Oc1ccc(OC(C(O)=O)C)cc1)ccc(C(F)(F)F)c2	3.12	6
521	CC(C(O)=O)Oc2ccc(Oc1c(Cl)cc(C(F)(F)F)cn1)cc2	2.9	6
522	OC(C3c2ccc(C(c1ccccc1)=O)n2CC3)=O	3.49	6
523	N(=O)(=O)c1c(Cl)ccc(OCC(O)=O)c1	2.96	6
524	OC(C2CC(=O)C(=C(C1CC1)O)C(=O)C2)=O	5.32	6
525	O=C(Oc(cc(c1)OC(OC(C2O)C(=O)O)C(O)C2O)c3c1)C=C3C	2.82	6
526	O=C(O)CCCCCN	4.43	6
527	O=C(O)CCCCCCCC=CCC=CCCCCC	4.77	6
528	O=C(O)C=C	4.26	6
529	O=C(O)C(O)C	3.79	6
530	O=C(O)C(=C)C	4.65	6
531	O=C(O)C(=CC)C	4.96	6
532	O=C(O)C(O)C(O)C(=O)O	3.06	6
533	O=C(O)C(Oc(c(cc(c1)Cl)C)c1)C	3.1	6
534	O=C(O)C=CC	4.17	6
535	O=C(O)CCCC	4.84	6
536	O=C(O)CCC(=O)O	4.21	6
537	O=C(O)C=CC(=O)O	3.03	6
538	O=C(O)CCCC(=O)O	4.34	6
539	O=C(O)CCCCCC	4.8	6
540	O=C(O)CCCCCC(=O)O	4.51	6
541	O=C(O)CCSCCC(=O)O	4.11	6
542	O=C(O)CCCCCCCC	4.95	6
543	O=C(O)CCCCCCCC(=O)O	4.55	6
544	O=C(O)CCCCC(=O)O	4.44	6
545	O=C(O)CCCCCCC	4.89	6
546	O=C(O)C(=O)C	2.45	6
547	O=C(O)C=Cc(cccc1)c1	4.44	6
548	O=C(O)CCCCC	4.88	6
549	O=C(O)CCCCCCCCCCC	5.3	6
550	O=C(O)C(=O)O	1.25	6
551	O=C(O)C(C(C(=O)O)C(O1)CC2)C12	3.4	6
552	O=CC(=O)O	3.3	6
553	c1c(O)c(O)ccc1C=CC(=O)O	4.62	6
554	CCCCCCCCCC(=O)O	4.9	6
555	c1cc(O)ccc1C=CC(=O)O	4.64	6
556	CC=CC(=O)O	4.17	6
557	O=C(O)CCCCCCC(=O)O	4.52	6
558	CC(=O)CC(O)=O	3.59	6
559	O=C(O)CCCCCCCCCCCCC	4.9	6
560	O=C(O)Cc(c(N(=O)=O)cc(N(=O)=O)c1)c1	3.5	6
561	c1cc(N(=O)=O)ccc1C=CC(=O)O	4.05	6

562	NCCCCCCC(=O)O	4.52	6
563	O=C(O)C=Cc(ccc(OC)c1)c1	4.54	6
564	NCCCCCCCC(=O)O	4.59	6
565	COc1ccccc1C=CC(=O)O	4.46	6
566	O=C(O)C=Cc(ccc(O)c1OC)c1	4.58	6
567	OC(=O)C(Cl)=C(Cl)Cl	1.15	6
568	COc1ccc(C=CC(=O)(O))cc1OC	4.53	6
569	O=CO	3.75	6
570	OCCCC(O)=O	4.72	6
571	OC(=O)CN(=O)=O	1.48	6
572	O=C(O)CCCCl	4.52	6
573	OC(=O)Cc1ccccc1Cl	4.07	6
574	BrCCCC(=O)O	4.58	6
575	OC(=O)CC=C	4.34	6
576	OC(=O)CCBr	3.99	6
577	CCC(O)C(O)=O	3.68	6
578	OC(=O)C(Cl)CC	2.84	6
579	O=C(O)C(Br)CC	2.55	6
580	O=C(O)C(F)F	1.24	6
581	Nc1cc(C(O)=O)ccc1	3.07	7
582	Nc1ccc(C(O)=O)cc1	2.38	7
583	Nc1cc(O)c(C(O)=O)cc1	2.05	7
584	Nc1ccc(O)cc1	5.48	7
585	Nc1cc(O)ccc1	4.37	7
586	Nc1cc(N(=O)=O)cc(N(=O)=O)c1	0.3	7
587	Nc1cc(Cl)cc(Cl)c1	2.51	7
588	Nc2ccc(c1ccccc1)cc2	4.35	7
589	Cc1c(N(=O)=O)ccc(N)c1	1.64	7
590	C(c1ccccc1)(=O)c2ccc(cc2)N	2.24	7
591	[N+](=O)([O-])c1c(C)cc(cc1C)N	2.54	7
592	[N+](=O)([O-])c1c(C)ccc(N)c1	0.4	7
593	S(C)(=O)(=O)c1ccc(cc1)N	1.35	7
594	N(=O)(=O)c1c(Cl)ccc(N)c1	1.9	7
595	C(F)(F)(F)c1ccc(cc1)N	2.45	7
596	C(OC)(=O)c1ccc(cc1)N	2.47	7
597	C(OCCCC)(=O)c1ccc(cc1)N	2.47	7
598	C(OCCC)(=O)c1ccc(cc1)N	2.49	7
599	C(OCC)(=O)c1ccc(cc1)N	2.51	7
600	c1(Cl)c(Cl)ccc(N)c1	2.97	7
601	C(F)(F)(F)c1cccc(N)c1	3.49	7
602	c1c(cccc1N)Br	3.58	7
603	c1c(cccc1N)I	3.61	7
604	c1(ccc(cc1)I)N	3.78	7
605	c1(ccc(cc1)Br)N	3.86	7
606	c1(C)cc(ccc1Br)N	4.05	7
607	c1(ccccc1N)C(O)=O	2.14	7
608	c1(ccccc1O)N	4.84	7
609	[N+](=O)([O-])c1ccc(c(N)c1)O	3.1	7

610	c1(Br)c(N)ccc(Br)c1	2.3	7
611	[N+](=O)([O-])c1cc(C)ccc1N	3.03	7
612	c1(ccccc1N)c2ccccc2	3.83	7
613	[N+](=O)([O-])c1cc(Cl)c(c(Cl)c1)N	-2.55	7
614	[N+](=O)([O-])c1cc(C)c(c(C)c1)N	0.98	7
615	c1(N)c(Cl)ccc(Cl)c1	2.05	7
616	c1(c(N)ccc(c1)[N+](=O)[O-])[N+](=O)[O-]	-4.25	7
617	[N+](=O)([O-])c1c(N)ccc(Cl)c1	-1.02	7
618	[N+](=O)([O-])c1ccc(c(Cl)c1)N	-0.94	7
619	c1(N)c(F)c(F)c(c(F)c1F)F	-0.28	7
620	c1(N)c(cccc1Cl)Cl	0.42	7
621	N(=O)(=O)c1c(N)ccc(OC)c1	0.77	7
622	[N+](=O)([O-])c1ccc(c(C)c1)N	1.04	7
623	c1(Cl)c(cccc1N)Cl	1.76	7
624	c1(Cl)c(N)ccc(Cl)c1	2	7
625	c1(ccccc1N)C(OCC)=O	2.18	7
626	c1(ccccc1N)C(OC)=O	2.23	7
627	[N+](=O)([O-])c1ccc(c(N)c1)C	2.35	7
628	c1(c(C)c(C)c(c(C)c1C)N)[N+](=O)[O-]	2.36	7
629	[N+](=O)([O-])c1ccc(c(N)c1)OC	2.49	7
630	c1(ccccc1Br)N	2.53	7
631	c1(ccccc1I)N	2.6	7
632	N(=O)(=O)c1cccc(c1N)N(=O)=O	-5	7
633	c1(N)c(Cl)cc(cc1Cl)Cl	-0.03	7
634	c1c(Cl)c(Cl)cc(Cl)c1N	1.09	7
635	c1(N)c(OC)ccc(OC)c1	3.93	7
636	N(=Nc(cccc1)c1)c(ccc(N)c2)c2	2.82	7
637	Nc(cccc1)c1	4.6	7
638	O=S(=O)(c(ccc(N)c1)c1)c(ccc(N)c2)c2	2.41	7
639	Nc(c(c(cc1)C)C)c1	4.7	7
640	Nc(c(ccc1)C)c1C	3.95	7
641	Nc(c(cc(c1)C)C)c1C	4.38	7
642	N(=O)(=O)c(c(N)ccc1)c1	-0.28	7
643	O(c(c(N)ccc1)c1)C	4.53	7
644	c(c(ccc1N)ccc2)(c2)c1	4.16	7
645	Nc(ccc(c(ccc(N)c1)c1)c2)c2	4.66	7
646	O(c(c(N)ccc1)c1)CC	4.43	7
647	Nc(c(ccc1)Cl)c1	2.66	7
648	Nc(c(ccc1)C)c1	4.44	7
649	Nc(c(N)ccc1)c1	4.47	7
650	Nc(ccc(c1C)C)c1	5.28	7
651	Nc(c(cc(c1)C)C)c1	4.89	7
652	Nc(c(cc(c1)Cl)C)c1	3.85	7
653	Nc(ccc(c1Cl)C)c1	4.05	7
654	Nc(c(ccc1C)C)c1	4.53	7
655	Nc1cc(cc(N(=O)=O)c1O)N(=O)=O	1	7
656	O=S(=O)(N)c(cccc1N)c1	2.9	7
657	O=C(c(cccc1N)c1)C	3.56	7

658	N(=O)(=O)c(cccc1N)c1	2.47	7
659	CC(C)c1ccc(N)cc1	4.85	7
660	N(=O)(=O)c(ccc(N)c1)c1	1	7
661	O(c(ccc(N)c1)c1)C	5.34	7
662	Nc(ccc(c1)Cl)c1	3.98	7
663	Nc(ccc(c1)C)c1	5.1	7
664	Nc(ccc(N)c1)c1	6.16	7
665	Nc(cccc1Cl)c1	3.52	7
666	Nc(cccc1C)c1	4.69	7
667	Nc(cccc1N)c1	4.98	7
668	Nc(cc(cc1C)C)c1	4.79	7
669	Nc(c(cc(c(ccc(N)c1C)c1)c2)C)c2	4.5	7
670	c(c(c(N)cc1)ccc2)(c2)c1	3.92	7
671	Cc1cc(C)c(N)cc1C	5.09	7
672	O(c(ccc(N)c1)c1)CC	5.2	7
673	Fc(c(N)ccc1)c1	3.2	7
674	Fc(c(cc(N)c1)Cl)c1	3.66	7
675	Fc(ccc(N)c1)c1	4.65	7
676	Nc1cccc(F)c1	3.5	7
677	c(c(c(N)cc1)c(N)cc2)(c1)c2	4.44	7
678	Nc1cc(OC)ccc1	4.24	7
679	O=C(c(c(N)ccc1)c1)C	2.22	7
680	Nc(c(ccc1)CC)c1	4.3	7
681	Nc(ccc(c1)CC)c1	5	7
682	Nc1c2cc3ccccc3cc2ccc1	4.1	7
683	Nc(c(ccc1)C(C)C)c1	4.42	7
684	c1cc(N)ccc1C(C)(C)C	4.95	7
685	N#Cc(ccc(N)c1)c1	1.74	7
686	S(c(cccc1N)c1)C	4	7
687	Nc1cccc(C#N)c1	2.75	7
688	Nc1cccc2c(N)cccc12	4.44	7
689	Nc1ccccc1SC	3.45	7
690	O=N(C(C=C2)=CC=C2NC1=CC=C(N)C=C1)=O	4.38	7
691	Nc1c2Cc3ccccc3c2ccc1	3.87	7
692	N(=Nc(cccc1)c1)c(ccc(N(C)C)c2)c2	2.96	7
693	c1ccccc1N2C(=O)C=C(C)N2C	1.4	7
694	N(c(c(c(ccc1)cc2)c1)c2)(C)C	4.83	7
695	N(c(cccc1)c1)(CC)CC	6.57	7
696	N(c(ccc(c1)C)c1)(C)C	5.63	7
697	N(c(ccc(N(C)C)c1)c1)(C)C	6.35	7
698	O=N(=O)c(ccc(N(C)C)c1)c1	0.61	7
699	N(c(cccc1)c1)C	4.85	7
700	N(c(cccc1C)c1)CC	5.25	7
701	N(c(cccc1)c1)CC	5.12	7
702	N(c(c(c(ccc1)cc2)c1)c2)CC	4.19	7
703	N(c(cccc1)c1)(C)C	5.15	7
704	N(c(cccc1C)c1)(C)C	5.34	7
705	N(c(cccc1)c1)c(cccc2)c2	0.78	7

706	OCCNc(cccc1)c1	4.06	7
707	O=C(N(c(cccc1)c1)C)C	-0.5	7
708	N(c(c(ccc1)C)c1)(C)C	5.94	7
709	N(c(c(ccc1)C)c1)C	4.62	7
710	O=N(=O)c(cccc1N(C)C)c1	2.63	7
711	N(c(ccc(c1)C)c1)C	5.36	7
712	N(c(cccc1C)c1)C	5	7
713	c1cc(OC)ccc1N(C)C	5.85	7
714	N(c(cccc1)c1)C(C)C	5.3	7
715	N(c(cccc1)c1)CCCC	5.12	7
716	CN(C)c1ccc(C#N)cc1	1.78	7
717	N(c(c(c(ccc1)cc2)c1)c2)C	3.67	7
718	c12ccccc1CCC(N)C2	9.93	8
719	c1(ccccc1)C(C)N	9.83	8
720	C(C(C)N)c1ccccc1	10.13	8
721	NCCC	10.71	8
722	NCCCC	10.78	8
723	NCC	10.87	8
724	NC1CCCCC1	10.63	8
725	NCc1ccccc1	9.33	8
726	c1(CN)ccc(cc1)C	9.36	8
727	c1(ccc(cc1)OC)CC(C)N	9.53	8
728	c12cc(ccc1OCO2)CC(C)N	9.67	8
729	NC(C)CCc1ccccc1	9.79	8
730	NCCCc1ccccc1	10.16	8
731	NCCCCc1ccccc1	10.36	8
732	NCC(C)C	10.68	8
733	c1(CN)ccco1	8.89	8
734	C(CN)(F)(F)F	5.7	8
735	c1(OC)c(OC)cc(cc1OC)CCN	9.56	8
736	C(CN)=C	9.7	8
737	NC(C)CC	10.56	8
738	NC	10.62	8
739	NC(C)C	10.63	8
740	C(C)(C)(C)N	10.68	8
741	NCCc1cnc2ccc(O)cc12	9.97	8
742	C1=NC=C(CCN)N1	9.8	8
743	NC(C)Cc(cccc1)c1	9.9	8
744	CC(N)C(O)c1cccc(O)c1	8.79	8
745	NC(=S)N	2.03	8
746	O=C(O)C(N)CC(c(c(N1)ccc2)c2)=C1	7.38	8
747	OCC(N)(CO)CO	8.07	8
748	OC(CN)C	9.94	8
749	OCC(N)CC	9.52	8
750	N(=O)(=O)c(ccc(NN)c1)c1	3.7	8
751	NCCN	9.92	8
752	NCCCN	10.62	8
753	NCCCCC	10.63	8

754	NCCCCN	10.8	8
755	NCCCCCC	10.64	8
756	NCCCCCCC	10.66	8
757	NCCCCCCCC	10.65	8
758	NCCCCCCCCC	10.64	8
759	OCC(N)(CO)C	8.8	8
760	OCC(N)(CC)CO	8.8	8
761	NC(CCCCC)C	10.58	8
762	NCCCCCCN	11.02	8
763	NCCCCCCCCCCCC	10.63	8
764	NCCCCCCCCCCCCCCCCCC	10.65	8
765	OCC(N)(C)C	10.19	8
766	OCCN	9.5	8
767	c1ccccc1CC(N)C	10.13	8
768	OCCCN	9.96	8
769	NN	7.96	8
770	C12(C)C3CCC4(C)C(C(=O)C)CCC4C3CCC1CC(N) CC2	9.18	8
771	N#CCN	5.34	8
772	c1c(Br)ccc(NN)c1	5.05	8
773	NC(C(N)CCC1)C1	10.24	8
774	C(#C)CN	8.15	8
775	n(c(ccc1)CCN)c1	10.03	8
776	n1c(CN)cccc1	9.09	8
777	NCc1ccc(N(=O)(=O))cc1	8.5	8
778	OC(c(cccc1)c1)C(N)C	9.44	8
779	CCCCC(C)CN	11.13	8
780	Cc(cccc1C)c1NC(C(CC)N)=O	8.71	8
781	O=S(=O)(N)c(ccc(c1)C)c1	10.17	8
782	c1cc(NC)ccc1S(=O)(=O)N	10.77	8
783	c1cc(S(=O)(=O)N)ccc1SCCO	9.27	8
784	c1cc(S(=O)(=O)N)ccc1S(=O)(=O)CCO	9.38	8
785	c1cc(SCCCO)ccc1S(=O)(=O)N	10.23	8
786	c1cc(S(=O)(=O)N)ccc1SCCC(C)(C)O	10	8
787	c1cc(S(=O)(=O)N)ccc1SCCCCCO	9.25	8
788	NS(=O)(=O)c1ccc(S(=O)(=O)CCCO)cc1	8.98	8
789	c1cc(S(=O)(=O)N)ccc1S(=O)(=O)CCC(C)(C)O	9.35	8
790	O=N(=O)c1cc(S(=O)(=O)N)ccc1SCCO	9.42	8
791	O=N(=O)c1cc(S(=O)(=O)N)ccc1SCCCO	9.38	8
792	O=N(=O)c1cc(S(=O)(=O)N)ccc1S(=O)(=O)CCCO	8	8
793	Nc1cc(S(=O)(=O)N)ccc1S(=O)(=O)CCCO	9.15	8
794	COC(=O)c1cc(S(=O)(=O)N)ccc1SCCCO	9.3	8
795	COC(=O)c1cc(S(=O)(=O)N)ccc1S(=O)(=O)CCCO	8.9	8
796	Fc1cc(S(=O)(=O)N)ccc1SCCO	9.76	8
797	Clc1cc(S(=O)(=O)N)ccc1SCCCO	9.45	8
798	Fc1cc(S(=O)(=O)N)ccc1SCCCO	9.64	8
799	Fc1cc(S(=O)(=O)N)ccc1SCCCCO	9.82	8
800	Fc1cc(S(=O)(=O)N)ccc1S(=O)(=O)CCO	9	8

801	Fc1cc(S(=O)(=O)N)ccc1S(=O)(=O)CCCO	9.02	8
802	NS(=O)(=O)c1ccc(c(F)c1)S(=O)(=O)CCCCO	9.6	8
803	c1cc(OC)ccc1C(=O)C2=CSC(S(=O)(=O)N)=C2	8.2	8
804	c1cc(O)ccc1C(=O)C2=CSC(S(=O)(=O)N)=C2	7.9	8
805	c1cc(OC)ccc1C(=O)C2=COC(S(=O)(=O)N)=C2	9.5	8
806	c1cc(C)ccc1C(=O)C2=CSC(S(=O)(=O)N)=C2	9.3	8
807	c1cc(OC)ccc1S(=O)(=O)C2=CSC(S(=O)(=O)N)=C2	8.78	8
808	C1COCCN1Cc2cc3cc(S(=O)(=O)N)oc3s2	9.2	8
809	c1cc(C)ccc1S(=O)(=O)C2=CSC(S(=O)(=O)N)=C2	8.95	8
810	c1cc(C)ccc1S(=O)(=O)C2=COC(S(=O)(=O)N)=C2	8.02	8
811	c1cc(OC)ccc1S(=O)(=O)C2=COC(S(=O)(=O)N)=C2	6.32	8
812	S1C(S(=O)(=O)N)=CC=C1S(=O)(=O)CCOC(=O)C	8.65	8
813	S1C(S(=O)(=O)N)=CC=C1SCCCCO	9.5	8
814	S1C(S(=O)(=O)N)=CC=C1S(=O)(=O)CCCCO	8.67	8
815	c1cc(S(=O)(=O)N)ccc1SCCCCO	10.17	8
816	c1cc(S(=O)(=O)N)ccc1S(=O)(=O)CCCCO	9.6	8
817	COC(=O)c1cc(S(=O)(=O)N)ccc1S(=O)(=O)CCO	9.2	8
818	c1cc(CCCO)ccc1S(=O)(=O)N	10.23	8
819	c1cc(S(=O)(=O)N)ccc1CCCOC(=O)C	10.22	8
820	c1cc(S(=O)(=O)N)ccc1CCCCO	10.32	8
821	c1cc(S(=O)(=O)N)ccc1CCCCCO	10.28	8
822	c1cc(S(=O)(=O)N)ccc1CCCCCOC(=O)C	10.3	8
823	CC(C)(C)CN	10.21	8
824	N(C)CCc1ccccc1	10.08	9
825	N(CC)CC	11.09	9
826	N1CCCCC1	11.28	9
827	N(CCCC)CCCC	11.39	9
828	N1CCOCC1	8.49	9
829	C(CNCC=C)=C	9.29	9
830	N(C)Cc1ccccc1	9.54	9
831	N(CC)Cc1ccccc1	9.64	9
832	N(C)C(C)Cc1ccccc1	9.87	9
833	c(ccc1CC(NC)C)cc1	9.87	9
834	N(C)CCCC	10.9	9
835	N(CCC)CCC	11	9
836	N1CCCCCC1	11.07	9
837	N(C(C)C)C(C)C	11.07	9
838	N1C(C)(C)CCCC1(C)C	11.72	9
839	N1CCCCC1C	11.08	9
840	N1CCC1	11.29	9
841	N1CCCC1	11.31	9
842	N1CCCCC1c2cccnc2	8.7	9
843	N1CCCC1c2ccccc2	9.4	9
844	N(C)C	10.73	9
845	N1CCCCC1CCC	11	9
846	C2CCC(NC1CCCCC1)CC2	10.4	9
847	CNCCCN2c1ccccc1CCc3ccccc23	10.4	9
848	c1cc2ccccc2cc1C(O)CNC(C)C	9.42	9

849	N(C1C)C1	8.22	9
850	OC(c(cccc1)c1)C(NC)C	10.25	9
851	N(C(CNC1C)C)C1	9.66	9
852	N(CCNC)C	10.16	9
853	N(CCNC1)C1	9.73	9
854	N(CC(C)C)CC(C)C	10.91	9
855	OC(C)CNCC(O)C	9.1	9
856	OCCNCCO	8.96	9
857	N(CCNCC)CC	11.06	9
858	C(#N)CCNCCC(#N)	5.26	9
859	N(C1)C1	8.04	9
860	CC(C)NCC(O)COc1cccc2ccccc12	9.42	9
861	O=S(=O)(O)CCCNC(CCCC1)C1	10.35	9
862	CCNNCC	7.71	9
863	N(C1)C1(C)C	8.64	9
864	c1ccccc1CS(=O)(=O)NC(=O)N	5.09	9
865	N(C(CC)C)C(CC)C	11.01	9
866	C1(NC(C)(C)C)CCCCC1	11.23	9
867	N1(CCOCC1)CC	7.67	10
868	c2c(CC(CN1CC(C)OC(C)C1)C)ccc(C(C)(C)C)c2	6.98	10
869	N(C)(C)CC	10.16	10
870	N(C)(C)CCCC	10.19	10
871	N1(C)CCOCC1	7.38	10
872	n1cccc(CN(C)C)c1	8	10
873	C(CN(CC=C)CC=C)=C	8.31	10
874	n1ccccc1CCN(C)C	8.75	10
875	n1cccc(c1)CCN(C)C	8.86	10
876	N(C)(C)Cc1ccccc1	8.91	10
877	C(c1ccccc1)(c2ccccc2)(C(CC)=O)CC(C)N(C)C	8.94	10
878	C(c1ccccn1)(c2ccc(cc2)Cl)CCN(C)C	9.13	10
879	N1(CCCC1)CCc2cccnc2	9.28	10
880	N1(CCCCC1)C	10.08	10
881	N1(CCCC1)C	10.32	10
882	N(CC)(CC)CC	10.78	10
883	N1(CCCCC1)CCC	10.41	10
884	CN1C(C)(C)CCCC1(C)C	11.25	10
885	N1(C)CCCC1c2cccnc2	8.18	10
886	CCN(CCCl)CCCl	6.57	10
887	C1(=CCCN(C)C1)C(OC)=O	7.16	10
888	c1(OCCN(C)C)cc(C)ccc1C(C)C	8.66	10
889	c1(C(C)C)c(OCCN(C)C)cc(c(OC(C)=O)c1)C	8.72	10
890	CC2N(CCCOC(c1cc(Cl)c(Cl)cc1)=O)CCCC2	8.9	10
891	c1(ccccc1C)C(OCCN(C)C)c2ccccc2	8.91	10
892	C(OCCN(C)C)(c1ccccc1)c2ccccc2	8.98	10
893	N(C)(C)C	9.8	10
894	CC(Cc1ccc(C(C)(C)C)cc1)CN2CCCCC2	10.1	10
895	N(CCC)(CCC)CCC	10.65	10
896	N(CCCC)(CCCC)CCCC	10.89	10

897	COC(=O)C1C(CC2CCC1N2C)OC(=O)c3ccccc3	8.61	10
898	CN(C)CCC=C2c1ccccc1CCc3ccccc23	9.4	10
899	CN(C)CCCN2c1ccccc1CCc3ccccc23	9.4	10
900	CCN(CC)CCNC(=O)c1ccc(N)cc1	9.32	10
901	CN1C2CC(CC1C3OC23)OC(=O)C(CO)c4ccccc4	7.75	10
902	O=C(OC(CC(N(C1C2)C)C2)C1)C(c(cccc3)c3)CO	9.43	10
903	CN(CCCl)CCCl	6.43	10
904	COc2ccc(CCN(C)CCCC(C#N)(C(C)C)c1ccc(OC)c(OC)c1)cc2OC	8.92	10
905	OC2(CCN(CCCC(=O)c1ccc(F)cc1)CC2)c3ccc(Cl)cc3	8.66	10
906	CCN(CC)CCCC(C)Nc1ccnc2cc(Cl)ccc12	10.1	10
907	CCOC(=O)C1(CCN(C)CC1)c2ccccc2	8.59	10
908	O=C(OCCN(CC)CC)c(ccc(N)c1)c1	8.05	10
909	c(ccc1c2CCN(C)C)cc1nc2	8.68	10
910	C1CCN(CC1)C2(CCCCC2)c3ccccc3	8.29	10
911	CCC(=O)OC1(CCN(C)CC1C)c2ccccc2	8.46	10
912	CCCCOc2cc(C(=O)NCCN(CC)CC)c1ccccc1n2	8.85	10
913	OCCN(CC)CC	9.87	10
914	N(CCC(C1)C2)(C1)C2	10.95	10
915	OCCN(CCO)CCO	7.76	10
916	OCCN(CCO)C	8.52	10
917	OCCN(C)C	9.31	10
918	N(CCN(C)C)(C)C	9.1	10
919	OC(C)CN(CC(O)C)CC(O)C	8.06	10
920	O=C(Nc(c(ccc1)C)c1C)CN(CC)CC	8.01	10
921	c1c(OC)c(OC)c(OC)cc1C(=O)NCc2ccc(OCCN(C)C)cc2	8.78	10
922	N(CCN(C1)C2)(C1)C2	8.82	10
923	CCN(CC)CCNC(=O)c1cc(Cl)c(N)cc1OC	9.27	10
924	ClCCN(CCCl)CCCl	4.64	10
925	CCCCN1CC1	7.86	10
926	O=S(=O)(O)CCCN(CCOC1)C1	7.15	10
927	c1ccccc1C(O)C(C)N(C)C	9.2	10
928	C1CCCCCCCCCCC1N2CC(C)OC(C)C2	8.08	10
929	OCC(N(C)C)(C)C	10.2	10
930	CN(O)C	5.2	10
931	O=P(C)(OCC)SCCN(C(C)C)C(C)C	9.12	10
932	CC(CC2)(C)CCC2(c3ccccc3)N1CCCCC1	8.27	10
933	CN1CCN(C(=O)c2ccccc2)CC1	6.78	10
934	C1CC(C(C)(C)C)CCC12(OCC(CN(CC)CCC)O2)	6.9	10
935	O1CCN(C)CC1(O)c2ccccc2	7.26	10
936	O1C(C)CN(C)CC1(O)c2ccccc2	7.41	10
937	O1CCN(C)CC1(O)c2ccc(Br)cc2	7.07	10
938	O1CCN(C)C(C)C1(O)c2ccccc2	7.88	10
939	O1C(c3ccccc3)C(C)N(C)CC1(O)c2ccccc2	7.68	10
940	O1CCN(C)C(c2ccccc2)C1(O)	6.02	10
941	O1CCN(C)CC1(O)c2cc(C(F)(F)F)ccc2	7.54	10
942	O1CCN(C)CC1(OCCC)c2ccccc2	7.12	10

943	O1CCN(C)CC1(OCC=C)c2ccccc2	7.09	10
944	O1CCN(C)C(C)C1(OCC)c2ccccc2	7.79	10
945	O1CCN(C)CC1(OCCCC)c2ccccc2	7.73	10
946	O1CCN(C)CC1(OCC(C)C)c2ccccc2	7.14	10
947	C(CCC1)N(C1)CC=C	9.7	10
948	N(C(CCCC1)C1)(C)C	10.7	10
949	CCCN(C)C	9.99	10
950	N(C(C)C)(C)C	10.3	10
951	N(CC(C)C)(C)C	9.91	10
952	C(C)(c1ccncc1)=O	3.59	11
953	n1cccc(c1)Br	2.91	11
954	C(OCC)(=O)c1cccnc1	3.35	11
955	C(OCC)(c1ccncc1)=O	3.45	11
956	n1ccc(cc1)Br	3.78	11
957	n1cccc(C=O)c1	3.8	11
958	n1ccc(cc1)Cl	3.84	11
959	n1ccc(cc1)C=O	4.77	11
960	n1ccc(cc1)c2ccncc2	4.82	11
961	C(OC)(=O)c1cccnc1	3.13	11
962	C(OC)(c1ccncc1)=O	3.26	11
963	n1cc(Cl)cc(Cl)c1	0.67	11
964	n1cccc(c1)Cl	2.84	11
965	n1cccc(c1)F	2.97	11
966	C(C)(=O)c1cccnc1	3.18	11
967	n1cccc(c1)I	3.25	11
968	n1cccc(CO)c1	4.9	11
969	n1ccc(cc1)CO	5.33	11
970	n1cccc(CCCO)c1	5.47	11
971	n1cccc(CC)c1	5.56	11
972	n1ccc(cc1)Cc2ccccc2	5.59	11
973	n1ccc(cc1)CCO	5.6	11
974	n1ccc(cc1)C=C	5.62	11
975	n1cccc(C)c1	5.63	11
976	n1ccc(cc1)CCCO	5.84	11
977	n1ccc(cc1)CC	5.87	11
978	n1ccc(cc1)C	5.98	11
979	C(C)(C)(C)c1ccncc1	5.99	11
980	n1ccc(cc1)CCC	6.05	11
981	n1cc(C)cc(C)c1	6.15	11
982	n1ccc(c(C)c1)C	6.46	11
983	n1ccc(cc1)OC	6.47	11
984	n1cccc(c1)CCN(C)C	4.3	11
985	N1CCCCC1c2cccnc2	3.21	11
986	N#Cc1ccncc1	1.9	11
987	N1(C)CCCC1c2cccnc2	3.1	11
988	C(=O)(N(CC)CC)c1cccnc1	3.5	11
989	C(=CC(O)=O)C(=O)c1cccnc1	3.82	11
990	n2cccc(c1ccccc1)c2	4.8	11

991	n1ccccc1	5.23	11
992	n1ccc(cc1)c2ccccc2	5.55	11
993	n1cccc(CN)c1	5.96	11
994	N(=O)(=O)c1cccnc1	1.18	11
995	n1cccc(O)c1	4.8	11
996	n1cccc(OC)c1	4.91	11
997	O=C(N)c(cccn1)c1	3.35	11
998	N#Cc(cccn1)c1	1.39	11
999	n(ccc(N)c1)c1	9.17	11
1000	ON=Cc1ccncc1	4.73	11
1001	n(ccc(N(C)C)c1)c1	10.14	11
1002	n1ccc(CN)cc1	4.39	11
1003	n1cc(NC(=O)C)ccc1	4.36	11
1004	Nc1ccncc1Br	7.05	11
1005	c1ccncc1CC(c2ccc(Cl)cc2Cl)=NOC	4.61	11
1006	n(cccc1N)c1	6	11
1007	n1cc(CC)ccc1C	6.51	12
1008	n1c(C)c(C)cc(C)c1C	7.9	12
1009	n1ccccc1F	-0.44	12
1010	n1ccccc1Cl	0.49	12
1011	n1ccccc1Br	0.9	12
1012	n1ccccc1C=C	4.98	12
1013	n1ccccc1Cc2ccccc2	5.13	12
1014	n1ccccc1CCO	5.31	12
1015	n1ccccc1CCCO	5.61	12
1016	n1cc(ccc1C)C=C	5.67	12
1017	C(C)(C)(C)c1ccccn1	5.76	12
1018	n1ccccc1CC	5.89	12
1019	n1cc(C)ccc1C	6.4	12
1020	n1cccc(C)c1C	6.57	12
1021	n1c(C)cccc1C	6.6	12
1022	n1ccc(cc1C)C	6.99	12
1023	n1c(C)cc(cc1C)C	7.43	12
1024	n1c(cccc1Cl)Cl	-2.86	12
1025	n1c(Cl)c(Cl)c(c(Cl)c1Cl)Cl	-1	12
1026	c1(Cl)c(cccn1)Cl	-0.85	12
1027	n1c(Cl)c(Cl)cc(Cl)c1Cl	-0.8	12
1028	n1ccccc1c2ccccc2	4.48	12
1029	n1ccccc1C	6	12
1030	n1ccccc1c2ccccn2	4.33	12
1031	n1ccccc1CO	4.86	12
1032	n1cc(C)ccc1N	7.22	12
1033	n1ccccc1SC	3.59	12
1034	n1ccccc1OC	3.06	12
1035	n1c(cccc1OC)OC	1.6	12
1036	n1ccccc1C(C)=O	2.73	12
1037	n1ccccc1C=O	3.8	12
1038	C(OC)(=O)c1ccccn1	2.21	12

1039	C(#N)c(nccc1)c1	-0.26	12
1040	n(c(N)ccc1)c1	6.86	12
1041	n1c(C(C)(C)C)cccc1C(C)(C)C	5.02	12
1042	ON=Cc1ccccn1	3.59	12
1043	O=N(=O)c(ccc(n1)N)c1	2.78	12
1044	c1cc(Cl)ncc1CN(C)C(C)=NC#N	0.7	12
1045	c1(Br)ncccn1	-1.63	13
1046	c1(ncccn1)C(OC)=O	-0.68	13
1047	c1(SC)ncccn1	0.59	13
1048	N(=O)(=O)c1cncnc1	0.72	13
1049	n1cccnc1	1.23	13
1050	c1(OCC)ncccn1	1.27	13
1051	n1cc(C)cnc1	1.91	13
1052	c1(SC)nc(C)cc(C)n1	2.12	13
1053	n1c(C)cc(nc1)C	2.7	13
1054	c1(N)ncccn1	3.45	13
1055	c2c(Nc1nc(C)cc(C)n1)cccc2	3.52	13
1056	c1(N)nc(C)cc(C)n1	4.82	13
1057	n1c(N)cc(nc1N)N	6.81	13
1058	CCc1nc(N)nc(N)c1c2ccc(Cl)cc2	7.34	13
1059	n1cnc(OC)cc1	2.5	13
1060	c1ccccc1Nc2nc(C3CC3)cc(C)n2	4.44	13
1061	c1(cn(cn1)C)[N+](=O)[O-]	-0.53	14
1062	c1(c[nH]cn1)[N+](=O)[O-]	-0.05	14
1063	c1(cncn1C)[N+](=O)[O-]	2.13	14
1064	n1(ccnc1)C(C)=O	3.6	14
1065	n1(ccnc1)C(COCCC)=Nc2ccc(cc2C(F)(F)F)Cl	3.7	14
1066	c2(c1ccccc1)[nH]ccn2	6.48	14
1067	n1ccn(C)c1	6.95	14
1068	n1cc[nH]c1	6.95	14
1069	n1cc[nH]c1C	7.85	14
1070	c1(cncn1C)CC(C(=O)O)NC(=O)CCN	7.04	14
1071	n1(ccnc1)C(=O)N(CCC)CCOc2c(Cl)cc(cc2Cl)Cl	3.8	14
1072	n2(C1CC(O)C(CO)O1)cnc3C(O)CNC=Nc23	5.2	14
1073	n2ccn(Cc1ccccc1)c2	6.7	14
1074	c1(cncn1C)CC2COC(=O)C2CC	6.78	14
1075	c1(CSCCN=C(NC)NC#N)[nH]cnc1C	6.8	14
1076	n1(ccnc1)CC(OCC=C)c2ccc(cc2Cl)Cl	6.53	14
1077	n2c1ccccc1[nH]c2	5.53	14
1078	COc3ccc(Cc2[nH]c1ccc(N(=O)=O)cc1n2)cc3	4.26	14
1079	Clc3ccc(Cc2[nH]c1ccc(Cl)cc1n2)cc3	4.86	14
1080	Cc3c(c2[nH]c1ccc(N(=O)=O)cc1n2)cccc3	4.87	14
1081	Cc3cc(C)c(c2[nH]c1ccc(N(=O)=O)cc1n2)cc3	5.29	14
1082	Clc3cc2nc(Cc1ccc(Br)cc1)[nH]c2cc3	5.42	14
1083	Cc3ccc(c2[nH]c1ccccc1n2)cc3	6.9	14
1084	Cc3ccc(Cc2[nH]c1ccc(Cl)cc1n2)cc3	7.09	14
1085	COc3c(c2[nH]c1ccccc1n2)cccc3	7.17	14
1086	Nc3ccc(Cc2[nH]c1ccc(Cl)cc1n2)cc3	7.47	14

1087	c1ccccc1CC2=NCCN2	10.3	14
1088	c1c(C)cc2N=C(c3c(OC)cc(OC)cc3)Nc2c1	7.25	14
1089	Cn1ccnc1Br	3.82	14
1090	Cn1ccnc1F	2.3	14
1091	Cn1ccnc1N	8.54	14
1092	C1=CN(C)C(N(=O)=O)=N1	-0.48	14
1093	Brc1ncc[nH]1	3.79	14
1094	Clc1ncc[nH]1	3.55	14
1095	CCc1ncc[nH]1	7.73	14
1096	C1=CN=C(N1)F	2.4	14
1097	N1C(N)=NC=C1	8.46	14
1098	N1C(N(=O)(=O))=NC=C1	-0.81	14
1099	CSC1=NC=CN1	5.95	14
1100	[O-][N+](=O)C1=CC2=C([NH]C=N2)C=C1	4.17	14
1101	ClC1=NC2=CC=CC=C2[NH]1	4.68	14
1102	C[N]1C=NC2=C1C=CC=C2	5.57	14
1103	CC1=CC2=C([NH]C=N2)C=C1	5.81	14
1104	CC1=CC2=C(C=C1C)N=C[NH]2	5.89	14
1105	CC1=NC2=CC=CC=C2[NH]1	6.1	14
1106	NC1=CC2=C([NH]C=N2)C=C1	6.11	14
1107	CCC1=NC2=CC=CC=C2[NH]1	6.2	14
1108	n2c1ccccc1nc2C(C)C	6.23	14
1109	n1cccc2ccccc12	4.9	15
1110	c12cccnc1cccc2O	5.02	15
1111	c12c(cccn1)ccc(OC)c2	5.03	15
1112	n2cccc1cc(OC)ccc12	5.03	15
1113	c12c(cccn1)cccc2C	5.05	15
1114	n2cc(C)cc1ccccc12	5.17	15
1115	n2cccc1cc(C)ccc12	5.34	15
1116	c12c(cccn1)ccc(C)c2	5.34	15
1117	n2cccc1cc(O)ccc12	5.15	15
1118	c12c(cccn1)ccc(O)c2	5.46	15
1119	c12ccccc1nccc2C	5.67	15
1120	n1c(C)ccc2ccccc12	5.71	15
1121	n2cc(Br)cc1ccccc12	2.69	15
1122	c12c(Cl)ccnc1cc(Cl)cc2	2.8	15
1123	c12c(cccn1)cccc2Cl	3.12	15
1124	c12c(cccn1)cccc2F	3.34	15
1125	n2cccc1cc(Cl)ccc12	3.85	15
1126	c12c(cccn1)ccc(Br)c2	3.87	15
1127	n2cccc1cc(Br)ccc12	3.87	15
1128	n1c(C)ccc2cc(C)ccc12	6.1	15
1129	n1c(C)cc(c2ccccc12)C	5.12	15
1130	n2cc(cc1ccccc12)O	4.28	15
1131	c12cccnc1c(ccc2Cl)O	3.56	15
1132	c12c(cccn1)cccc2OC	5.01	15
1133	n1c(C)ccc2cccc(O)c12	5.55	15
1134	c12c(cccc1c(ccn2)C)O	5.56	15

1135	CCCCCC(OC(COc2c1ncccc1c(Cl)cc2)=O)C	3.75	15
1136	COc3ccc(Cc1nccc2cc(OC)c(OC)cc12)cc3OC	8.07	15
1137	c1ccc2c(c1)ccc3ncccc23	4.21	15
1138	c1ccc2c(c1)ccc3cccnc23	4.21	15
1139	n(c(c(ccc1)cc2)c1N)c2	3.95	15
1140	c1ccc2c(N)ccnc2c1	9.13	15
1141	Nc2ccc1ncccc1c2	6.61	15
1142	n(c(c(ccc1)cc2)c1)c2N	7.3	15
1143	c1c(N)c2cccnc2c(O)c1	5.67	15

Table S2. Optimal hyperparameters for each model after dimension reduction

Model	Hyperparameter type	Hyperparameter range	Optimal hyperparameters
Catboost	iterations	100 - 1200	657
	learning_rate	0.01 - 0.3	0.1006443961371804
	depth	4 - 10	9
	l2_leaf_reg	1 - 10	2.108946494987559
XGBoost	n_estimators	300 - 2500	338
	learning_rate	0.01 - 0.3	0.12085361159092464
	max_depth	4 - 12	9
	min_child_weight	1 - 10	3
	reg_alpha	0.0 - 10.0	8.640448069013212
	reg_lambda	0.1 - 10.0	5.023173051570108
	subsample	0.6 - 1.0	0.8466267905361617
	colsample_bytree	0.6 - 1.0	0.9832300822748906
GBDT	n_estimators	100 - 1200	785
	learning_rate	0.01 - 0.3	0.01406163546546719
	max_depth	4 - 10	6
	min_samples_split	2 - 20	5
	min_samples_leaf	1 - 10	8
	subsample	0.6 - 1.0	0.9190907092018442
RF	n_estimators	100 - 1200	392
	max_depth	4 - 20	20
	min_samples_split	2 - 20	3
	min_samples_leaf	1 - 10	2
	max_features	0.2 - 1.0	0.5402252163402858

Table S3. The specific models constructed using B-MF and C-MF respectively

ML algorithm	Molecular fingerprint type	Train set		Val set		Test set	
		R^2	*RMSE*	R^2	*RMSE*	R^2	*RMSE*
Catboost	B-MF	0.909	1.254	0.806	1.783	0.845	1.615
	C-MF	0.955	0.645	0.823	1.180	0.880	1.073
XGBoost	B-MF	0.901	1.308	0.822	1.708	0.850	1.585
	C-MF	0.959	0.616	0.841	1.117	0.891	1.030
GBDT	B-MF	0.922	1.160	0.832	1.660	0.839	1.644
	C-MF	0.924	0.839	0.838	1.128	0.876	1.088
RF	B-MF	0.863	1.536	0.841	1.614	0.833	1.676
	C-MF	0.933	0.790	0.820	1.187	0.859	1.160

Table S4. Domain parameters of the Catboost

Model	Test set	
	N	R^2
without AD_{SAL}	228	0.891
with AD_{SAL} $\{\rho_{s,T} \geq 1.000, I_{A,T} \leq 1.025\}$	62	0.926

Table S5. Experimental and predicted values of pK_a for compounds within the external validation domain and their application domain parameters

Num	SMILES	Exp.pK_a	Pred.pK_a	$\rho_{s,T}$	$I_{A,T}$
1	ClC1(=C(C(=CC=C1)C)C(=O)O)	2.59	2.92335	2.4653334715586146	0.7413490726286734
2	ClC1(=C(C(=O)O)C=C(C)C=C1)	3.12	3.49267	3.0955752467603492	0.7624057833864144
3	ClC1(=C(C(=O)O)C=CC(=C1)C)	3.27	3.49267	3.0955752467603492	0.7624057833864144
4	ClC1(=C(C(=O)O)C=CC(=C1)Cl)	2.74	2.80199	3.95739297870431	0.8137497910999208
5	ClC1(=C(C(=O)O)C=CC(=C1)[N+](=O)[O-])	1.96	2.29828	4.967689510226641	0.7378250799574746
6	ClC1(=C(C(=O)O)C=CC=C1)	2.95	3.17027	3.886245909417892	0.7542128643094623
7	ClC1(=C(C(=O)O)C=CC=C1C)	3	3.40274	2.426590928900194	0.6773248818756156
8	ClC1(=C(C(C(=O)O)=CC=C1)C)	3.43	3.42392	2.2978931217635967	0.7201582547412435
9	ClC1(=C(C([N+](=O)[O-])=CC=C1)C(=O)O)	1.41	1.70809	4.549575781030791	0.8355772477077535
10	ClC1(=C(C=C(C(=O)O)C=C1)C)	4.07	3.95822	3.8083032144484514	0.8202731357103692
11	ClC1(=C(C=C(O)C(=C1)C(C)C)C)	9.98	9.79564	1.1191306525084552	0.6109012084355855
12	ClC1(=C(C=C(O)C=C1)C)	9.4	9.49908	2.9379805733579865	0.9659002062448039
13	ClC1(=C(C=CC(=C1)C(=O)O)C)	4.06	3.95822	3.8083032144484514	0.8202731357103692
14	ClC1(=C(O)C(=CC(=C1)Cl)C)	7.85	6.98155	1.080617920521325	0.9819229649885605
15	ClC1(=C(O)C(=CC=C1)C)	8.7	8.51784	2.374074767697918	1.0053416165032485
16	ClC1(=C([N+](=O)[O-])C=C(C(=O)O)C=C1)	3.33	2.95904	5.041525990452166	0.8398242020406352
17	ClC1(=C([N+](=O)[O-])C=CC=C1C(=O)O)	2.02	2.06496	4.2965484848342665	0.7527579663863794
18	ClC1(=CC(=C(C(=O)O)C=C1)C)	3.75	3.4246	2.9335022026969906	0.7248799084915216
19	ClC1(=CC(=C(C)C=C1)C(=O)O)	3.63	3.4246	2.9335022026969906	0.7248799084915216
20	ClC1(=CC(=C(O)C=C1)C)	9.6	9.4388	2.962501469817786	1.002032124830005
21	ClC1(=CC(C(=O)O)=CC=C1)	3.8	3.6645	3.0121165668990626	0.6658917947157598

22	ClC1(=CC(O)=CC=C1)	8.96	9.18133	2.5218339497800644	0.9574598014964155
23	ClC1(=CC([N+](=O)[O-])=CC(=C1)C(=O)O)	2.17	2.90711	1.3049007831382817	0.7889740215599242
24	ClC1(=CC=C(C(=O)O)C=C1)	3.98	3.7118	2.26461524374886	0.6757572902317036
25	ClC1(=CC=C(O)C=C1)	9.29	9.33386	2.1742108144062	0.7878853195411011
26	ClCC(=O)O	2.85	2.95265	1.2167673987580598	0.6429446288219522
27	ClCC1(=CC=C(C(=O)O)C=C1)	4.13	4.31111	1.4815054732279584	0.6163212156159565
28	ClCCC(=O)O	4.04	3.79506	1.629918692928069	0.47427055179600053
29	FC1(=C(C(=O)O)C=C([N+](=O)[O-])C=C1)	2.73	2.64926	2.3372822262997337	0.6985942681659564
30	FC1(=C(C(=O)O)C=CC=C1)	3.6	3.42033	3.065853681400122	0.6388622996008356
31	FC1(=C([N+](=O)[O-])C=C(C(=O)O)C=C1)	3.37	3.01141	2.493560306666397	0.7844654871349387
32	FC1(=CC(C(=O)O)=CC=C1)	3.9	3.80426	3.740262505646013	0.47502782935472726
33	FC1(=CC=C(C(=O)O)C=C1)	4.16	3.89773	3.4309782604601033	0.5244951277256765
34	FC1(=CC=C(O)C=C1)	9.88	9.16858	1.91926657257505	0.9361296805485284
35	FCC(=O)O	2.56	2.79333	1.0760620629362085	0.5051109912014278
36	IC1(=C(C(=CC=C1)C)C(=O)O)	2.7	3.18187	1.6759230746082365	0.6287972996494283
37	IC1(=C(C(=O)O)C=C(C)C=C1)	2.95	3.78806	1.911857486833249	0.6475470797910619
38	IC1(=C(C(=O)O)C=CC(=C1)C)	3.2	3.78806	1.911857486833249	0.6475470797910619
39	IC1(=C(C(=O)O)C=CC=C1)	2.93	3.42981	3.68758463019469	0.6915692392338559
40	IC1(=C(C(=O)O)C=CC=C1C)	2.92	3.67976	2.0193687254089907	0.6273527266492951
41	IC1(=C(C(C(=O)O)=CC=C1)C)	3.26	3.67976	1.6348999412708558	0.6196604543508412
42	IC1(=CC(=C(C(=O)O)C=C1)C)	3.79	3.71241	2.1669528340781588	0.6449995489936255
43	IC1(=CC(=C(C)C=C1)C(=O)O)	3.62	3.71241	2.1669528340781588	0.6449995489936255
44	IC1(=CC(C(=O)O)=CC=C1)	3.83	3.78767	3.3525104605166325	0.5136461194472536

45	IC1(=CC(O)=CC=C1)	9.02	9.44514	1.3065015178476367	0.9717394902297363
46	IC1(=CC=C(C(=O)O)C=C1)	3.99	3.87962	2.5033154858310773	0.5766089812360738
47	IC1(=CC=C(O)C=C1)	9.32	9.61259	1.8570644595790768	0.8093897284024134
48	ICC(=O)O	3.16	3.25228	1.0758276208868631	0.4170297268696817
49	O(C1(=C(O)C=CC=C1))C	9.7	9.82432	1.3825068947237533	0.9718720881901881
50	O=C(C1(=C(O)C=CC=C1))C	10.37	7.96153	1.738557249807498	0.9595122488877982
51	O=C(C1(=CC(=CC=C1)C(C(=O)O)C))C2(=CC=CC=C2)	4.2	4.13739	1.5344716293198442	0.6283127516497072
52	O=C(C1(=CC(O)=C(O)C=C1))C	7.8	7.95859	1.0875293511404966	0.8282890039300776
53	O=C(C1(=CC(O)=CC=C1))C	9.12	8.11735	1.936277462539203	0.86766317644797
54	O=C(C1(=CC(OC)=C(O)C=C1))C	7.65	8.38081	1.1214772162613942	0.7719011817214638
55	O=C(C1(=CC=C(O)C=C1))C	7.9	8.28278	3.223296685080319	0.9565731682507472
56	O=C(NC1(=CC(O)=CC=C1))C	9.38	8.3113	1.0240978241174394	1.0143810694241682
57	O=C(NO)C=1(C=2(C(C=CC=1)=CC=CC=2))	7.7	5.13683	1.3801136294266718	0.8314155622185432
58	O=C(O)C(C)(C)C	5.04	4.55223	1.013829810943735	0.955119442607319
59	O=C(O)C(C)C	4.76	3.91922	1.1170607432039625	0.42873087622735684
60	O=C(O)C(C1(=CC=C(CC(C)C)C=C1))C	4.57	5.24637	1.0984029988570703	0.9248667511295564
61	O=C(O)C(O)(C1(=CC=CC=C1))C	3.53	3.64833	1.272406478801154	0.4732167042534716
62	O=C(O)C(O)(C1(=CC=CC=C1))C2(=CC=CC=C2)	3.05	2.98888	1.4435145245407304	0.8402399578838096
63	O=C(O)C(O)C1(=CC=CC=C1)	3.375	3.48926	1.398225730666191	0.6321799752974904
64	O=C(O)C1(=C(C(=CC=C1)C)C)	3.73	3.94709	3.5545687374169015	0.6352072432364053
65	O=C(O)C1(=C(C(=O)C)C=CC=C1)	4.15	3.42937	2.681044184944151	0.6141899940161138
66	O=C(O)C1(=C(C(=O)N)C=CC=C1)	3.75	3.56289	1.7381141442853953	0.8161751294300261
67	O=C(O)C1(=C(C(O)=CC=C1)C)	3.83	3.76229	3.682638448269623	0.6922209674283144

68	O=C(O)C1(=C(C(OC)=CC=C1)C)	3.72	3.80532	2.135951259640525	0.5353969702470441
69	O=C(O)C1(=C(C=C(C)C=C1)C)	4.18	4.05858	4.073662655017143	0.6855714120126793
70	O=C(O)C1(=C(C=C(O)C=C1)C)	4.71	4.09368	3.7404477030784333	0.698026812360738
71	O=C(O)C1(=C(C=C(OC)C=C1)C)	4.54	3.99952	2.288168024696986	0.603540725834749
72	O=C(O)C1(=C(C=C(OCC)C=C1)C)	4.54	3.89559	1.1388508881027581	0.596765074055459
73	O=C(O)C1(=C(C=CC(=C1)C)C)	3.97	4.05858	4.073662655017143	0.6855714120126793
74	O=C(O)C1(=C(C=CC(=C1)O)C)	3.92	4.09368	3.7404477030784333	0.698026812360738
75	O=C(O)C1(=C(C=CC(=C1)OC)C)	3.84	3.99952	2.288168024696986	0.603540725834749
76	O=C(O)C1(=C(C=CC(=C1)OCC)C)	3.86	3.89559	1.1388508881027581	0.596765074055459
77	O=C(O)C1(=C(C=CC=C1)C(C)(C)C)	3.53	4.35753	1.0242467514523657	0.9163113541196977
78	O=C(O)C1(=C(C=CC=C1)C(C)C)	3.64	3.90234	1.1699066192664365	0.8551025892601013
79	O=C(O)C1(=C(C=CC=C1)C)	3.92	3.79349	4.985660807025933	0.6678678474249303
80	O=C(O)C1(=C(C=CC=C1)C=O)	4.56	3.79684	1.5350961395425133	0.7277857923558737
81	O=C(O)C1(=C(C=CC=C1)CC)	3.79	3.736	2.9580851589738724	0.6217788253069224
82	O=C(O)C1(=C(C=CC=C1)CO)	3.84	3.47757	1.863659683372013	0.6790737945183458
83	O=C(O)C1(=C(C=CC=C1)COC)	3.85	4.43962	1.7822734311196464	0.5841395785863536
84	O=C(O)C1(=C(C=CC=C1C)C)	3.23	3.45752	3.330184388774227	0.7003471184121287
85	O=C(O)C1(=C(O)C(=CC=C1)C)	3.02	3.31468	3.944968444166863	0.6618114147441047
86	O=C(O)C1(=C(O)C=C(C)C=C1)	3.11	3.71073	5.273047246760705	0.721218129752164
87	O=C(O)C1(=C(O)C=CC(=C1)C)	3.01	3.71073	5.273047246760705	0.721218129752164
88	O=C(O)C1(=C(O)C=CC=C1)	2.99	3.77789	4.1564937280704495	0.7933649053642022
89	O=C(O)C1(=C(O)C=CC=C1C)	3.35	3.44258	3.768057654571004	0.6930446730342761
90	O=C(O)C1(=C(OC)C(=CC=C1)C)	3.84	3.80532	2.192541079156064	0.5630605754079389
91	O=C(O)C1(=C(OC)C=C(C)C=C1)	4.38	4.01572	2.434743329948828	0.6080985983342517
92	O=C(O)C1(=C(OC)C=CC=C1)	4.05	3.60574	3.8892277093204637	0.5437535843747996

93	O=C(O)C1(=C(OC)C=CC=C1C)	3.46	3.29155	2.3531069645223734	0.5556548121015803
94	O=C(O)C1(=C(OCC)C=CC=C1)	4.18	3.50744	2.462707533898584	0.5266367373463368
95	O=C(O)C1(=C(OCC)C=CC=C1C)	3.51	3.32212	1.0660669163868268	0.5496478001597292
96	O=C(O)C1(=C(OCCC)C=CC=C1)	4.24	4.32885	1.5377277775508547	0.5479588832603987
97	O=C(O)C1(=C([NH3+])C=CC=C1)	2.08	3.42981	2.301572558344338	0.7485670215912847
98	O=C(O)C1(=C([NH3+])C=CC=C1C)	3.2	3.18187	1.4843202973650105	0.6390431523000464
99	O=C(O)C1(=CC(=C(C)C=C1)C)	4.41	4.30574	3.9664499738650996	0.7097758925568395
100	O=C(O)C1(=CC(=C(O)C=C1)C)	4.68	4.5798	4.570385241914307	0.7676669422703796
101	O=C(O)C1(=CC(=C(OC)C=C1)C)	4.35	4.24306	3.23596746475711	0.5908113282142394
102	O=C(O)C1(=CC(=CC(=C1)C)C)	4.3	4.28492	1.7803618569342317	0.7530437483208804
103	O=C(O)C1(=CC(=CC=C1)C(C)C)	4.27	4.12391	1.0311357177547515	0.6423462921512696
104	O=C(O)C1(=CC(=CC=C1)C)	4.28	4.12497	4.810100831476087	0.5717211721450176
105	O=C(O)C1(=CC(C#N)=CC=C1)	3.6	3.313	2.2775853813613334	0.4679779695852816
106	O=C(O)C1(=CC(C(=O)C)=CC=C1)	3.84	3.87926	1.7941922228015543	0.5785487347554115
107	O=C(O)C1(=CC(O)=C(C)C=C1)	4.32	4.5798	4.570385241914307	0.7676669422703796
108	O=C(O)C1(=CC(OC)=C(C)C=C1)	4.13	4.24306	3.23596746475711	0.5908113282142394
109	O=C(O)C1(=CC(OC)=C(O)C(=C1)OC)	4.2	4.35417	1.3379177281461996	0.4519083037537297
110	O=C(O)C1(=CC(OC)=CC=C1)	4.06	3.86775	3.7746855105012918	0.5293383167513444
111	O=C(O)C1(=CC(OCC)=CC=C1)	4.17	3.78447	2.4482305988159365	0.5077762006564321
112	O=C(O)C1(=CC(OCCCC)=CC=C1)	4.25	4.66535	1.109565711026006	0.7254197167892841
113	O=C(O)C1(=CC([NH3+])=CC=C1)	3.09	3.78767	2.085830292905028	0.5782852979115023
114	O=C(O)C1(=CC=C(C#N)C=C1)	3.53	3.24985	1.522520836162959	0.5316923394670443
115	O=C(O)C1(=CC=C(C(=O)C)C=C1)	3.72	3.9766	1.6654193070604024	0.6520277513334412
116	O=C(O)C1(=CC=C(C)C=C1)	4.37	4.29386	4.099767775329905	0.6063490449886649
117	O=C(O)C1(=CC=C(CO)C=C1)	4.18	4.23833	1.6421966799706977	0.6259462947963552

118	O=C(O)C1(=CC=C(COC)C=C1)	4.18	5.06156	1.7082292930768332	0.6697821024470165
119	O=C(O)C1(=CC=C(C[NH3+])C=C1)	3.59	4.39544	1.428072126922196	0.6057774071401587
120	O=C(O)C1(=CC=C(O)C=C1)	4.57	4.64252	3.647895138550658	0.767904463592177
121	O=C(O)C1(=CC=C(OC)C=C1)	4.45	3.98472	2.9889795932225445	0.6019218412708879
122	O=C(O)C1(=CC=C(OCC)C=C1)	4.64	3.93388	3.049602547353965	0.6447895322374256
123	O=C(O)C1(=CC=C(OCCC)C=C1)	4.63	4.62591	2.445936261516816	0.7530110079554839
124	O=C(O)C1(=CC=C(OCCCC)C=C1)	4.48	4.79048	1.7696809222998384	0.9858796371862035
125	O=C(O)C1(=CC=C(OCCCCC)C=C1)	4.49	4.84247	1.1551983496277933	0.997365757807445
126	O=C(O)C1(=CC=CC=C1)	4.18	3.72197	3.0185374759749406	0.6149206165745965
127	O=C(O)C1(CCCCC1)	4.89	4.89496	1.1669839375171485	0.28517725586106474
128	O=C(O)C2(=C(C(=O)C1(=CC=CC=C1))C=CC=C2)	3.54	3.52735	1.6298908688729985	1.0013334227574635
129	O=C(O)C2(=C(C1(=CC=CC=C1))C=CC=C2)	3.46	3.64104	2.760205384929876	0.8835655734311468
130	O=C(O)C2(=C(OC1(=CC=CC=C1))C=CC=C2)	3.53	3.586	2.333271404637959	0.7696210802676682
131	O=C(O)C2(=CC(OC1(=CC=CC=C1))=CC=C2)	3.95	3.90659	1.4392291592221402	0.7419897490951065
132	O=C(O)C2(=CC=1(C(=CC=CC=1)C=C2))	4.2	3.98385	3.2460435065232938	0.7233127073427758
133	O=C(O)C2(=CC=C(OC1(=CC=CC=C1))C=C2)	4.52	3.85763	1.0054693206164793	0.7342777810459079
134	O=C(O)C2(=[NH+]C=1(C(=CC=CC=1)C=C2))	1.79	3.51772	1.1031762969610308	0.6980369851424031
135	O=C(O)C=1(C(O)=CC2(=CC=CC=C2(C=1)))	2.71	3.59831	1.2427208321424297	0.7757894217753186
136	O=C(O)C=1(C=2(C(C=CC=1)=CC=CC=2))	3.7	3.52168	3.5327744189056407	0.6971569634256927
137	O=C(O)C=1(C=2(C(C=CC=1C)=CC=CC=2))	3.11	3.33795	2.356975317607239	0.6893638985396741

138	O=C(O)C=1(C=3(C(C =C2(C=1C=CC=C2)) =CC=CC=3))	3.65	3.60767	2.1029034 878861963	0.6841475217 568589
139	O=C(O)C=2(C1(=C(C =CC=C1)NC=2))	5.27	4.33719	1.3392493 435428023	0.8417225642 375168
140	O=C(O)C=2(C=1(C(= CC=C(C=1)O)C=CC =2))	3.69	4.29608	1.4266401 98185701	0.7746624949 649772
141	O=C(O)C=3(C=1(C(= CC2(=CC=CC=C2(C =1)))C=CC=3))	3.7	3.66145	1.6262981 633780287	0.7099763659 673106
142	O=C(O)C=C	4.25	3.56696	1.0378160 84741767	0.2265909370 5226963
143	O=C(O)C=CC1(=C(C =CC=C1)C)	4.5	4.31153	1.0048661 042523768	0.4942164310 845832
144	O=C(O)C=CC1(=C(O)C=CC=C1)	4.56	4.34313	1.0021710 563423047	0.5549402556 861798
145	O=C(O)C=CC1(=C(O C)C=CC=C1)	4.42	4.12555	1.8848295 203730445	0.3885271853 5635083
146	O=C(O)C=CC1(=CC(O)=CC=C1)	4.4	4.83855	1.2160099 920339493	0.4870510757 1500705
147	O=C(O)C=CC1(=CC(OC)=CC=C1)	4.38	4.02728	1.5357527 814452772	0.4073811545 0109363
148	O=C(O)C=CC1(=CC =C(C)C=C1)	4.56	4.53244	1.1435611 602119202	0.4656881549 369145
149	O=C(O)C=CC1(=CC =C(O)C=C1)	4.36	4.92035	1.8064377 5251784	0.4994233660 432291
150	O=C(O)C=CC1(=CC =C(OC)C=C1)	4.315	4.1979	1.8655622 754439707	0.4053558016 448202
151	O=C(O)C=CC1(=CC =CC=C1)	4.14	4.00807	1.5343425 14679802	0.3994553387 197797
152	O=C(O)C=O	1.26	3.49783	1.0186050 853706705	0.4239273873 4147184
153	O=C(O)CC	4.83	3.72621	1.2588222 777837368	0.3414320588 889943
154	O=C(O)CC#N	2.46	2.28518	1.0208953 200529884	0.4486989520 451072
155	O=C(O)CC(=O)C	3.09	3.56647	1.3375281 118372138	0.6373221959 38035
156	O=C(O)CC(C)C	4.79	4.22124	1.3485711 118776504	0.1249148986 0268401
157	O=C(O)CC(O)C	4.5	3.65805	1.1061657 984772348	0.2751111624 205653
158	O=C(O)CC1(=CC=C(OC)C=C1)	4.36	4.21175	2.0774772 65544247	0.4578667348 6985036
159	O=C(O)CC1(=CC=C([NH3+])C=C1)	3.49	4.09879	1.3104169 238153087	0.4560051441 175847
160	O=C(O)CC1(=CC=C C=C1)	4.27	3.81315	2.0613073 29867522	0.5315453006 704578

161	O=C(O)CC1(CCCCC1)	4.8	5.03599	1.159344115638123	0.18932811192052218
162	O=C(O)CC2(=CC=1(C(=CC=CC=1)C=C2))	4.26	4.06626	2.555794982228513	0.6259059024373813
163	O=C(O)CC2(=CC=C(C1(=CC=CC=C1))C=C2)	4.46	4.08555	1.0840187585442889	0.8020225842931243
164	O=C(O)CC=1(C=2(C(C=CC=1)=CC=CC=2))	4.24	3.78798	2.242593563084053	0.6239576874183562
165	O=C(O)CC=2(C1(=C(C=CC=C1)NC=2))	4.39	4.59054	1.6901377819854109	0.9013229526324792
166	O=C(O)CC=C	4.33	3.72301	1.044944833612943	0.28407806406251346
167	O=C(O)CCC	4.8	4.55684	1.8061970855157143	0.12359362656455075
168	O=C(O)CCC(=O)C	4.63	4.37951	1.3448347129296117	0.5566420506180425
169	O=C(O)CCC(C)C	4.84	5.09294	1.283647526606433	0.08388978341399715
170	O=C(O)CCC1(=C(OC)C=CC=C1)	4.8	4.82254	1.3057141529748633	0.3959087038668714
171	O=C(O)CCC1(=CC(OC)=CC=C1)	4.65	4.98673	1.2304981832917454	0.40168956281400325
172	O=C(O)CCC1(=CC=C(C)C=C1)	4.74	5.34162	1.2473190644656866	0.5235523747554743
173	O=C(O)CCC1(=CC=C(OC)C=C1)	4.72	5.1424	1.1024211530573755	0.47461529448489803
174	O=C(O)CCC1(=CC=CC=C1)	4.59	4.76082	2.1299384049989127	0.5689848007892019
175	O=C(O)CCCC	4.82	4.80598	2.0271727763217116	0.11103279493595747
176	O=C(O)CCCC1(=CC=CC=C1)	4.78	4.95566	1.7243401172653898	0.6134759957193562
177	O=C(O)CCCCC	4.85	4.88089	1.656972489189613	0.1357175171601947
178	O=C(O)CCCCC1(=CC=CC=C1)	4.88	4.9708	1.6918886935745094	0.674906906046047
179	O=C(O)CCCCCC	4.86	4.92962	2.1482738222722206	0.1629449373555221
180	O=C(O)CCCCCCCCC	4.89	4.98697	1.191004762459528	0.2596976453474489
181	O=C(O)CCCCCCCCCCC	4.89	4.98329	1.324300104544179	0.33198527291259117
182	O=C(O)COC1(=C(C=CC=C1)C)	3.17	3.53456	3.9749672569177474	0.3000761938772005
183	O=C(O)COC1(=C(C=CC=C1C)C)	3.36	3.63049	1.0726335133806546	0.4851148109820802

184	O=C(O)COC1(=CC(=CC=C1)C)	3.18	3.5896	3.82504504198469	0.2891040381359881
185	O=C(O)COC1(=CC=C(C)C=C1)	3.15	3.82289	3.088451691858613	0.36503068753313345
186	O=C(O)COC1(=CC=CC=C1)	3.15	3.1088	3.2009259941147032	0.30505338278390287
187	O=C(OC)C1(=C(O)C=CC=C1)	10.3	7.44391	1.3011506431562707	0.83413007130073
188	O=C(OC)C1(=CC=C(O)C=C1)	8.34	7.80859	1.6022033217211131	1.006760050607582
189	O=C([O-])C1(=C(O)C=C(C)C=C1)	13.76	7.76859	1.24119430753784	0.8694375436824977
190	O=C([O-])C1(=C(O)C=CC(=C1)C)	13.96	7.76859	1.24119430753784	0.8694375436824977
191	O=C([O-])C1(=CC=C(O)C=C1)	9.35	7.84785	1.1080784177580414	0.8929297237416266
192	O=C1(C(C(=O)O)=CC=CC=C1)	3.73	3.4125	1.813516046961176	0.7122902003095397
193	O=C1(C(O)=CC(C)=CC=C1)	7.26	8.52744	1.259283743604152	1.0006595052627025
194	O=C1(C=CC(C(=O)O)=CC=C1)	3.2	3.79116	1.7168252291713766	0.6282017117059167
195	O=CC1(=C(O)C(OC)=CC=C1)	7.91	7.73604	1.4256366164035539	0.8975083837083883
196	O=CC1(=C(O)C=CC=C1)	8.36	8.33052	1.7085773291791413	0.8510394965944945
197	O=CC1(=CC(O)=CC=C1)	8.86	8.49212	1.6253297116859744	0.8009151382745542
198	O=[N+]([O-])C1(=C(C(=CC=C1)C)C(=O)O)	2.13	2.2892	3.2867125922650136	0.8480812146578289
199	O=[N+]([O-])C1(=C(C(=O)O)C=C(C)C=C1)	2.55	3.06796	3.558159184016977	0.8628665683454796
200	O=[N+]([O-])C1(=C(C(=O)O)C=CC(=C1)C)	2.68	3.06796	3.558159184016977	0.8628665683454796
201	O=[N+]([O-])C1(=C(C(=O)O)C=CC=C1)	2.23	2.55501	4.28615665653739	0.7659337897918753
202	O=[N+]([O-])C1(=C(C(=O)O)C=CC=C1C)	2.91	2.93438	2.544132008851561	0.7827217186268909
203	O=[N+]([O-])C1(=C(C(C(=O)O)=CC=C1)C(=O)C)	3.26	1.898	1.6292703288984671	0.770310469243848
204	O=[N+]([O-])C1(=C(C(C(=O)O)=CC=C1)C)	2.98	2.8588	3.153929834653134	0.7932260339108341
205	O=[N+]([O-])C1(=C(C(O)=CC=C1)C(=O)O)	2.24	2.31905	4.106209544508529	0.8448377330710523

206	O=[N+]([O-])C1(=C(C=C(C(=O)O)C=C1)C)	3.65	3.43526	4.103135969167363	0.8842435413704199
207	O=[N+]([O-])C1(=C(C=CC(=C1)C(=O)O)C)	3.62	3.43526	4.103135969167363	0.8842435413704199
208	O=[N+]([O-])C1(=C(O)C(C(=O)O)=CC=C1)	2.16	1.97979	4.146451589310213	0.7746040840707739
209	O=[N+]([O-])C1(=C(O)C(C(=O)[O-])=CC=C1)	10.27	9.62175	1.3700190173868085	0.9999210379200184
210	O=[N+]([O-])C1(=C(OCC(=O)O)C=CC=C1)	2.9	2.57322	3.1021251088813204	0.4097643810488638
211	O=[N+]([O-])C1(=CC(=C(C(=O)C)C=C1)C(=O)O)	3.27	2.64767	1.242771010731619	0.7418779528503615
212	O=[N+]([O-])C1(=CC(=C(C(=O)O)C=C1)C(=O)C)	3.06	2.64767	1.242771010731619	0.7418779528503615
213	O=[N+]([O-])C1(=CC(=C(C(=O)O)C=C1)C)	2.91	3.07021	3.4408902828519	0.8602375196833184
214	O=[N+]([O-])C1(=CC(=C(C)C=C1)C(=O)O)	3.17	3.07021	3.4408902828519	0.8602375196833184
215	O=[N+]([O-])C1(=CC(=C(CO)C=C1)C(=O)O)	3.14	2.59987	1.664808059679206	0.7104071852700451
216	O=[N+]([O-])C1(=CC(=C(O)C=C1)C(=O)O)	2.15	2.56207	4.195099181844528	0.8048770547773281
217	O=[N+]([O-])C1(=CC(=CC=C1)C=CC(=O)O)	3.855	3.29795	1.306108175842735	0.6036695442718234
218	O=[N+]([O-])C1(=CC(C(=O)O)=CC=C1)	3.47	3.09946	4.260429110959235	0.6752266631530853
219	O=[N+]([O-])C1(=CC(O)=C(C(=O)O)C=C1)	1.83	2.56207	4.195099181844528	0.8048770547773281
220	O=[N+]([O-])C1(=CC=C(C(=O)C)C=C1)	16.65	4.72531	1.045817428256372	0.9554756365220476
221	O=[N+]([O-])C1(=CC=C(C(=O)O)C=C1)	3.43	3.24359	3.6976499834080356	0.6774212888302225
222	O=[N+]([O-])C1(=CC=C(CCC(=O)O)C=C1)	4.54	4.51836	1.4255392977608987	0.6682306808298323

223	O=[N+]([O-])C1(=CC=C(OCC(=O)O)C=C1)	2.89	2.90548	3.174219432354254	0.4214684821653672
224	O=[N+]([O-])CC(=O)O	1.63	2.40017	1.0107132087968695	0.7328242936647634
225	OC1(=C(C=C(O)C=C1)C)	10.11	10.10428	1.7218496571103987	1.0058187708675195
226	OC1(=C(C=CC=C1)C)	10.22	10.02799	3.8337157504452657	0.9748808930224999
227	OC1(=C(O)C=CC(=C1)C)	9.62	10.00041	1.7132082038959977	1.0155947988583782
228	OC1(=C(O)C=CC=C1)	9.33	9.27704	2.25434625753352	1.0149205182541212
229	OC1(=C(O)C=CC=C1C)	9.28	9.60981	1.6403239120491235	1.005109885448168
230	OC1(=C2(C(=CC=C1)CCC2))	10.2	10.06473	1.7371643314015162	0.10465960640883351
231	OC1(=C2(C(=CC=C1)CCCC2))	10.46	10.09246	1.8274841068971992	0.09568582872448947
232	OC1(=CC(=CC=C1)C)	10.01	10.06578	2.9595989258814264	1.0091320694030175
233	OC1(=CC=C(C(C)(C)C)C=C1)	10.27	10.58496	1.0677363020072237	0.9468984277322544
234	OC1(=CC=C(C)C=C1)	10.22	10.30716	1.9900693464321484	1.0061056740820054
235	OC1(=CC=C(CC(C)(C)C)C=C1)	10.71	10.65413	1.274449235625255	0.6083456957416975
236	OC1(=CC=C(O)C=C1)	9.93	9.60635	1.1122812688499188	0.9641415858665489
237	OC2(=CC1(=C(CCCC1)C=C2))	10.33	10.55048	1.3150790620988397	0.1509062457697449
238	OC=2(C1(=NC=CC(=C1C=CC=2)C))	8.44	5.39482	2.1516306105325183	0.9774636407659725
239	OC=3(C=1(C(=CC2(=CC=CC=C2(C=1)))C=CC=3))	9.88	8.27329	1.1246832230298638	0.9618254082781911
240	S(C1(=C(C(=O)O)C=CC=C1))C	3.89	3.68883	1.732082931340546	0.6992238788092339
241	S(C1(=C([N+](=O)[O-])C=CC=C1))CC(=O)O	3.18	2.72977	1.0537307916796497	0.6575320167723079
242	S(C1(=CC([N+](=O)[O-])=CC=C1))CC(=O)O	3.4	2.96247	1.1034941984097473	0.5776407167387626
243	S(C1(=CC=C(C(=O)O)C=C1))C	4.23	4.05854	1.4114224804420652	0.6461596349745544
244	S(C1(=CC=C([N+](=O)[O-])C=C1))CC(=O)O	3.2	3.1448	1.1577224454893282	0.6270538059149193

245	S1(C(C(C(=O)O)N3(C1C(NC(=O)CC2(=CC=CC=C2))C3=O))(C)C)	2.76	3.57557	1.053377246192367	0.04157496866250023
246	S2(C1(=C(C=CC=C1)C(=C2)C(=O)O))	4.24	3.63212	1.0018416346305172	0.7260197927528802
247	SC1(=CC(C(=O)O)=CC=C1)	3.96	3.78767	2.085830292905028	0.5782852979115023
248	SC2(=CC=1(C(=NC=CC=1)C=C2))	6.5	4.48103	1.6621504744603977	0.7551480326688204
249	[Br]C(C(=O)O)C	2.97	3.12869	1.0988009307089333	0.5703851583485877
250	[Br]C1(=C(C(=CC=C1)C)C(=O)O)	2.71	3.14329	2.0708661930963803	0.6321861899419434
251	[Br]C1(=C(C(=O)O)C=C(C)C=C1)	3	3.58309	2.280672544062684	0.6479168436479187
252	[Br]C1(=C(C(=O)O)C=CC(=C1)C)	3.09	3.58309	2.280672544062684	0.6479168436479187
253	[Br]C1(=C(C(=O)O)C=CC=C1)	2.93	3.14384	3.6449575656845354	0.7044484677542144
254	[Br]C1(=C(C(=O)O)C=CC=C1C)	2.9	3.37435	2.2016466532569137	0.6271324643800056
255	[Br]C1(=C(C(C(=O)O)=CC=C1)C)	3.36	3.51193	1.8804540295667111	0.6371447005170727
256	[Br]C1(=C(C([N+](=O)[O-])=CC=C1)C(=O)O)	1.37	1.70725	3.111108206932289	0.7889818974453366
257	[Br]C1(=C(C=C(C(=O)O)C=C1)C)	4.03	3.80582	3.015412209013795	0.6355728762438451
258	[Br]C1(=C(C=CC(=C1)C(=O)O)C)	3.96	3.80582	3.015412209013795	0.6355728762438451
259	[Br]C1(=C([N+](=O)[O-])C=C(C(=O)O)C=C1)	3.24	2.66169	2.850439286362141	0.7904097500931295
260	[Br]C1(=CC(=C(C(=O)O)C=C1)C)	3.77	3.36986	2.4887511978304695	0.6439294580597119
261	[Br]C1(=CC(=C(C)C=C1)C(=O)O)	3.58	3.36986	2.4887511978304695	0.6439294580597119
262	[Br]C1(=CC(C(=O)O)=CC=C1)	3.79	3.43767	3.6649446321115	0.5469628091738145
263	[Br]C1(=CC(O)=CC=C1)	8.99	8.89083	2.3054066270195706	0.9885062303401391
264	[Br]C1(=CC=C(C(=O)O)C=C1)	3.99	3.54168	3.2880755162622184	0.569800381579057
265	[Br]C1(=CC=C(O)C=C1)	9.29	8.97781	2.1908849232390155	0.8790692176638326
266	[Br]CC(=O)O	2.84	2.95671	1.0767663105347745	0.4053739018705605

267	[Se](C1(=C([N+](=O)[O-])C=CC=C1))CC(=O)O	3.45	2.72977	1.0539919778647437	0.6574528422601202
268	[Se](C1(=CC([N+](=O)[O-])=CC=C1))CC(=O)O	3.55	2.96247	1.103435666026435	0.577648477324733
269	[Se](C1(=CC=C([N+](=O)[O-])C=C1))CC(=O)O	3.46	3.1448	1.156386694016736	0.6271873324403994
270	ClC2(=CC=C(C(C1(=NC=CC=C1))CCN(C)C)C=C2)	9.16	8.53277	1.0620772739001554	0.8590675518923037
271	FC1(=C(N)C=CC=C1)	2.96	3.04803	1.987930981358768	0.9291174000257867
272	IC1(=CN=CC=C1)	3.25	3.9739	1.5393601515334312	1.0038309492037103
273	N#CC1(=CC=NC=C1)	1.91	2.01549	1.4302286982248522	0.8983001444163484
274	N(C(C)C)C(C)C	11.04	10.54446	1.0107185189950363	0.08516721329517604
275	N(C(CC1(=CC=CC=C1))C)C	9.87	9.87	1.6382725463224934	0.669453523394135
276	N(C)(C)C	9.96	9.39893	1.0118084185603358	0.5739644568627699
277	N(C)C	10.98	9.85029	1.0003354780580855	0.2192761893658705
278	N(C1(=C(C=CC=C1)C))C	4.59	5.63481	1.5517149201774219	0.9620977857143429
279	N(C1(=CC(=CC=C1)C))C	4.94	4.97583	1.8765432054213158	0.6560272195756992
280	N(C1(=CC=C(C)C=C1))C	5.36	5.35785	1.5713866586472869	0.6639703573673967
281	N(C1(CCCCC1))(C)C	10.48	10.26081	1.013499629018847	0.48805888746942033
282	N(C=1(C=2(C(C(=CC=1)=CC=CC=2)))(C)C	4.83	5.34523	1.5494828438700525	0.8699629563379953
283	N(C=1(C=2(C(C(=CC=1)=CC=CC=2)))C	3.7	4.71296	1.9708882981148683	0.8029344194943038
284	N(C=1(C=2(C(C(=CC=1)=CC=CC=2)))CC	4.19	4.97741	1.7273878190455545	0.7327523188719278
285	N(CC(C)C)(C)C	9.91	10.19514	1.012217823325294	0.16222349266300612
286	N(CC(C)C)CC(C)C	10.59	10.88082	1.0112203080037845	0.09850091783006898
287	N(CC)(C)C	10.05	9.61231	1.1607870393076218	0.11938300173568703
288	N(CC)(CC)CC	10.78	10.47665	1.0283244340159436	0.5093689872272226

289	N(CC=C)CC=C	9.35	8.94095	1.0000062570346426	0.1396268845874011
290	N(CCC)(C)C	9.62	9.83472	1.5650346549897065	0.1695085443813106
291	N(CCCC)(C)C	10.03	10.16013	1.4801828927348795	0.1678301621203161
292	N(CCCC)(CCCC)CCCC	10.8	10.14211	1.002300036562864	0.6146552382352752
293	N(CCN(C)C)(C)C	9.15	9.70149	1.0291118557481598	0.48326225808611323
294	N(CCNC)C	10.11	10.45554	1.0005704384514087	0.29696555301715083
295	N1(=C(C(=CC=C1)C)C)	6.57	6.13225	1.3137059020046702	0.7735626667820815
296	N1(=C(C=C(C)C=C1)C)	6.77	6.46046	1.368443079314643	0.9112699406821965
297	N1(=C(C=CC=C1C)C)	6.85	5.5724	1.2790598897408891	0.9640394704921719
298	N1(=CC(=CC(=C1)C)C)	6.15	5.83243	1.114372908479502	0.9883499762806923
299	N1(=CC(=CC=C1)C)	5.81	5.4727	1.8573679835077797	0.93353988857922
300	N1(=CC(=CC=C1)CC)	5.57	5.88405	2.0074840201654327	0.6848325829192852
301	N1(=CC=C(CC)C=C1)	5.94	5.62661	1.9501604432025599	0.6076096125651638
302	N1(C(CCCC1(C)C)(C)C)	11.17	11.3553	1.0452101588024	0.31825089441082016
303	N1(C(CCCC1(C)C)(C)C)(C)	11.25	10.97583	1.0439388320339578	0.29046285076981887
304	N1(C(CNC(C1)C)C)	9.87	9.67985	1.006738025522975	0.7199992791768577
305	N1(CCCC1)(C)	10.58	10.11808	1.000484821088196	0.6276938770164356
306	N1(CCCCC1)	11.24	10.60967	1.5326634045116148	0.2759734618031612
307	N1(CCCCCC1)	10.99	10.60967	1.4620777642235963	0.21143437744727997
308	N12(CCN(CC1)CC2)	8.75	9.45553	1.0000479919283987	0.5035064010001923
309	N2(=CN(CC1(=CC=CC=C1))C=C2)	6.66	6.13839	1.0799157884784603	0.9032268521169997
310	N2(C1(=C(C=CC=C1)CCC3(=C2C=CC=C3)))(CCCNC)	10.65	9.61922	1.279325181960289	0.7684100359220168
311	N=1(C=2(C(C(C)=CC=1)=CC=CC=2))	5.51	5.79574	3.000786378129475	0.9233155254259373
312	N=1(C=2(C(C=C(C=1)C)=CC=CC=2))	5.15	5.31163	2.2995165048862276	0.6956479812506227

313	N=1(C=2(C(C=CC=1)=CC=CC=2))	4.89	4.67293	1.8196883916486901	0.9677433483537894
314	N=1(C=2(C(C=CC=1)=CC=CC=2C))	4.87	4.92385	3.186525023534716	0.7886037214938477
315	N=1(C=2(C(C=CC=1C)=CC=CC=2))	5.69	5.5732	2.4529419540781063	0.8818650532605014
316	N=2(C1(=C(C=CC(=C1)N)NC=2))	6.11	6.55159	1.1814750087989572	0.7986146633513724
317	N=2(C1(=C(C=CC=C1)N(C=2)C))	5.65	5.22857	1.1750617754641146	0.5322652406413227
318	N=2(C1(=C(C=CC=C1)NC=2C))	6.3	6.16754	1.4925459415586972	0.7791054439444832
319	N=2(C1(=C(N)C=CC=C1C=C3(C=2C=CC=C3)))	4.4	5.4388	1.0838235122258921	0.9476080331662887
320	N=2(C1(=C3(C(=CC=C1C=CC=2)C=CC=C3)))	4.25	4.26814	1.8630888786557933	0.8775061451569451
321	N=2(C=1(C(=C(C=CC=1)C)C=CC=2))	5	5.79574	3.000786378129475	0.9233155254259373
322	N=2(C=1(C(=C(N)C=CC=1)C=C3(C=2C=CC=C3)))	6.04	6.30964	1.2102846837981418	0.9647728801876421
323	N=2(C=1(C(=CC(C)=CC=1)C=CC=2))	5.1	5.62493	2.7906297982420125	0.6888039463595513
324	N=2(C=1(C(=CC=C(C=1)C)C=CC=2))	5.25	5.62493	2.7906297982420125	0.6888039463595513
325	N=3(C=2(C(=C1(C(C=CC=C1)=CC=2))C=CC=3))	5.15	4.96069	1.3465536657008828	0.9310687090280443
326	NC	10.76	9.20358	1.0000000529632895	0.03180116497722114
327	NC(C)(C)C	10.73	10.07704	1.0243358061410248	0.33513655009025717
328	NC(C)C	10.64	10.1149	1.0068562129650618	0.04254883209251437
329	NC(C1(=CC=CC=C1))C	9.36	8.21901	1.938704194080087	0.5498496954318863
330	NC(CC1(=CC=CC=C1))C	9.9	10.27752	3.853343027184633	0.3267842963851072
331	NC(CCC1(=CC=CC=C1))C	9.78	10.14856	2.3261930238035027	0.3411389248332343
332	NC(CCCC1(=CC=CC=C1))C	9.99	10.32512	1.4609329308448653	0.3723466369611387
333	NC(CCCCC)C	10.975	11.07597	1.1116407639191506	0.3486200658525655
334	NC1(=C(C=CC=C1)CC)	4.37	4.60237	1.0363846177041536	1.01551327584286
335	NC1(=C(N)C=CC=C1)	4.43	3.71542	1.9477048635915954	0.9860971938256778

336	NC1(C(N)CCCC1)	9.795	10.30896	1.0267035335084504	0.2546236266901518
337	NC1(CCCCC1)	10.67	10.47426	1.0547923694865529	0.894775322004718
338	NC=1(C=2(C(C=CC=1)=CC=CC=2))	3.92	3.9848	2.7778722486278156	1.0164642298565152
339	NCC(C)(C)C	10.17	10.48019	1.023809884401527	0.28223679302002663
340	NCC(C)C	10.36	10.5131	1.0242746184399192	0.3552441857975834
341	NCCC	10.65	10.37175	1.3205379723972839	0.068770966807332
342	NCCCC	10.7	10.68901	1.5021056199825935	0.09903740435229927
343	NCCCCC	10.65	10.73817	1.120336670327527	0.14538576810961581
344	NCCCCCC	10.68	10.78691	1.7090231120058794	0.17823598001135132
345	NCCCCCCC	10.34	10.69978	1.7006220333002924	0.2590456540897819
346	NCCCCCCN	10.97	10.24496	1.1480430691859256	0.6838971125859716
347	O(C1(=C(N)C=CC=C1))C	4.51	3.96861	1.2310943209365632	1.0195109416882049
348	O(C1(=C(N)C=CC=C1))CC	4.51	4.58143	1.5470439054085403	0.9448461954433798
349	O(C1(=CN=CC=C1))C	4.89	4.92988	1.7594325811921752	0.9854979299816266
350	O(C1(=NC=CC=2(C1=CC=CC=2)))C	3.05	4.79699	1.7945160821410884	0.6713864364395471
351	O(C2(=CC=1(C(=NC=CC=1)C=C2)))C	5.05	5.06385	3.2933794095961098	0.4597090721393807
352	O(C2(=NC=1(C(=CC=CC=1)C=C2)))C	3.17	5.10428	1.2558451691812715	0.7871755143061056
353	O(C=1(C=2(C(N=CC=1)=CC=CC=2)))C	6.45	5.2843	1.8703166784889338	0.7368078521945344
354	O(C=2(C1(=NC=CC=C1C=CC=2)))C	4.88	4.70479	2.4757391176218846	0.5878705822348851
355	O=C(C1(=CC(N)=CC=C1))C	3.61	3.68859	1.8117243862211905	1.0177071227630583
356	O=C(C1(=CN=CC=C1))C	3.32	3.56762	1.7399575865219084	0.6798659244392805
357	O=C(C1(=NC=CC=C1))C	-0.46	-0.89393	1.381931427949901	0.959386194992311
358	O=C(N1(CCN(C)CC1))C2(=CC=CC=C2)	6.78	7.02691	1.0357826005959485	0.9784282768315133
359	O=C(NC1(=CN=CC=C1))C	4.44	3.8026	1.398254691078411	0.8048593263285467
360	O=C(OC)C1(=CC(N)=CC=C1)	3.55	2.59018	1.4514447700315656	0.9282214492049137

361	O=C(OC)C1(=CC=NC=C1)	3.38	3.44234	1.6189365991435016	0.6302313378586948
362	O=C(OC)C1(=CCCN(C1)C)	7.55	7.10068	1.000212789045269	0.7651860988179656
363	O=C(OC)C1(=CN=CC=C1)	3.23	3.02403	1.8139837289756944	0.5826203136148652
364	O=C(OC)C2(=CC=1(C(=NC=CC=1)C=C2))	3.8	3.49397	1.0107108671465315	0.5835401999920348
365	O=C(OCC)C2(C1(=CC=CC=C1))(CCN(C)CC2)	8.6	7.79237	1.0744882876890933	0.7989610196111293
366	O=C1(OCC(C1CC)CC=2(N(C=NC=2)C))	7.08	6.35088	1.000002407171986	0.5211708801684533
367	O=CC1(=CC=NC=C1)	4.63	4.18042	1.1984814420876677	0.7106810152472022
368	OC(C1(=CC=CC=C1))C(N)C	8.81	8.95972	1.6314989040284151	0.5575379679250314
369	OC(C1(=CC=CC=C1))C(NC)C	9.676666667	8.83672	1.2944632567346162	0.8567928646694982
370	OCC(N)(CO)CC	8.8	9.63962	1.0000469092500477	0.5658674514664885
371	OCC1(=CC=NC=C1)	5.41	4.7631	1.0675292704209254	0.5612868372206195
372	OCC1(=CN=CC=C1)	5	4.78445	1.7390288138923689	0.6569435832197006
373	OCCC1(=CC=NC=C1)	5.68	5.57116	2.1104284394360873	0.3616228436516573
374	OCCC1(=CN=CC=C1)	5.38	5.6265	1.6474482892015214	0.49462181748593803
375	OCCC1(=NC=CC=C1)	5.37	5.14641	1.0848594758978571	0.9722680319308553
376	OCCCC1(=CC=NC=C1)	5.9	5.88277	2.1662833666666836	0.34121320460965565
377	OCCCC1(=CN=CC=C1)	5.57	5.91307	1.9322379859138739	0.4206678043163542
378	OCCCC1(=NC=CC=C1)	5.71	5.43297	1.4574589957208461	0.7824299417688945
379	OCCCCC1(=CC=NC=C1)	6.01	5.95279	1.2771865766339534	0.34324511478856107
380	OCCCCC1(=CN=CC=C1)	5.7	5.9282	1.12436841583847	0.41807228475791786
381	OCCCN	10	9.90534	1.3028357306361629	0.3717382630747194
382	OCCN	9.55	9.58549	1.2893486978590396	0.370571937293736
383	OCCN(CC)CC	9.84	9.83236	1.028957326109279	0.5522520475902952
384	OCCN(CCO)CCO	7.76	7.77025	1.0015753858457686	0.6959197507701753

385	OCCNCCO	9.04	8.61543	1.0016300012595636	0.6433923815889386
386	S(C1(=NC=CC=2(C1=CC=CC=2)))C	3.93	4.86082	1.015281960783104	0.914302705319575
387	S(C2(=CC=1(C(=NC=CC=1)C=C2)))C	4.75	5.0908	1.241955844963215	0.6825776003811412
388	[Br]C=1(C=NC2(=CC=CC=C2(C=1)))	-0.44	-0.46194	1.6461455993037908	0.7334641161214929
389	[S-]C=2(C1(=NC=CC=C1C=CC=2))	8.29	8.82417	1.4382819403096558	0.9475434809994469
390	[SH2+]C2(=CC=1(C(=NC=CC=1)C=C2))	3.95	4.48103	1.6621504744603977	0.7551480326688204

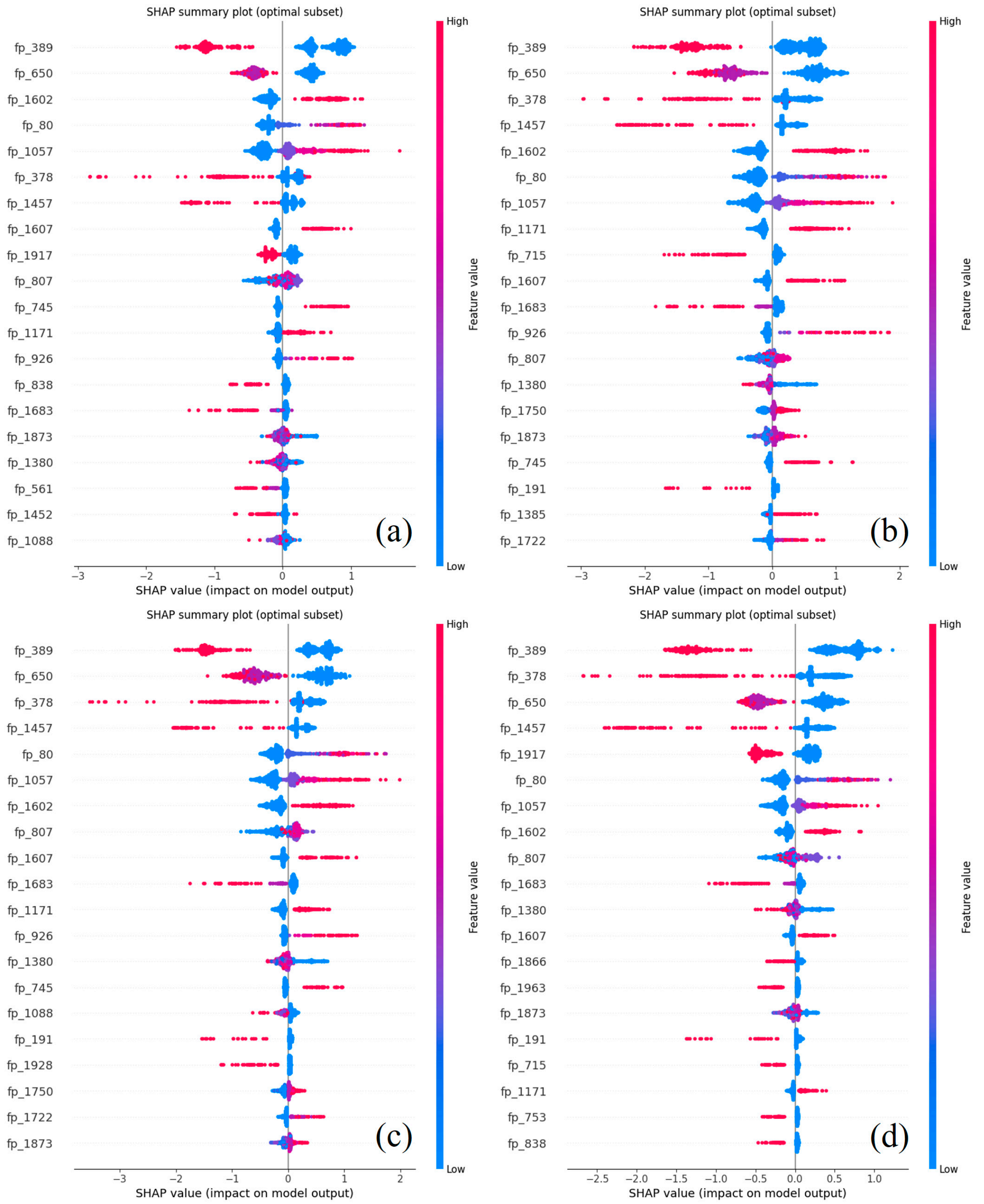

Figure S1. SHAP maps of the top 20 features with the highest SHAP contributions for each model after dimensionality reduction: (a) Catboost; (b) XGBoost; (c) GBDT; (d) RF

Text S1. Use RDKit to identify the structure corresponding to the molecular fingerprint

First, a reference set of molecular structures was generated from the unique SMILES within the dataset. For each high-ranking fingerprint bit, its encoded atomic environment was extracted and mapped to the corresponding local molecular substructure within the reference set. Finally, these substructures were translated into standardized SMARTS pattern representations to enable clear and consistent identification of key functional groups and structural motifs.

To enable intuitive chemical interpretation, the decoded substructures corresponding to high-importance fingerprint bits were visualized using the RDKit toolkit. Within an exemplary molecular scaffold from the dataset, the specific atoms constituting each key substructure were highlighted in red. These visualizations were systematically assembled into a composite figure, with each panel clearly annotated by the feature's SHAP rank, its fingerprint bit index, and the corresponding SMARTS pattern. Furthermore, a summary table was constructed to document the mapping results for all high-contributing features, including instances where a clear substructure match could not be identified.

This interpretability workflow established a direct, reproducible link between the abstract, high-dimensional fingerprint features and their concrete chemical correlations. By doing so, it verified the chemical rationality of the high-SHAP-value descriptors and provided the foundational mapping necessary for the subsequent quantitative analysis of how specific functional group properties—such as electron-withdrawing or donating ability—govern pKa variation. The entire procedure was integrated into the model construction pipeline, ensuring consistency and reproducibility.

Text S2. Details on ADSAL $\{\rho_{s,q} \geq \rho_{s,T}, I_{A,q} \leq I_{A,T}\}$ characterization

An AD characterization method, AD_{SAL} $\{\rho_{s,q} \geq \rho_{s,T}, I_{A,q} \leq I_{A,T}\}$ (abbreviated as AD_{SAL}) was adopted in the current study, with similarity density (ρ_s) to characterize chemical space of training data, and inconsistency of activity (I_A) to warn the emergence of ACs. $\rho_{s,T}$ and $I_{A,T}$ are the corresponding thresholds. ρ_s defines weighted similarity density between a query compound and training compounds:

$$\rho_{s,q} = \sum t \in T^{w_{q,t,e}} \quad (1)$$

where the subscript q and t represent a query compound and a training compound; T represents the entire training set; and $w_{q,t,e}$ stands for weight function for the pair of compound q and t, calculated as

$$w_{q,t,e} = e^{\frac{-a\times(1-S_{M,q,t})}{S_{M,q,t}+\varepsilon}} \quad (2)$$

where $S_{M,q,t}$ stands for pairwise molecular similarity between compound q and t, which were calculated with Morgan fingerprints; a is a parameter that modulates weighted contribution of training compounds, which was set to 10; ε is an infinitesimal quantity to ensure that the denominator is not zero, which was set to 1×10^{-6} .

In the current study, I_A was calculated to quantify weighted inconsistency of activity between a query compound (q) and training compounds ($t \in T$):

$$I_{A,q} = \frac{\sum t{\in}T^{w_{q,t,e}} \cdot S_{WD,t}}{\sum t{\in}T^{w_{q,t,e}}} \quad (3)$$

where $S_{WD,t}$ stands for weighted local discontinuity scores (S_{WD}) for the training compound (t). $S_{WD,t}$ was proposed to be calculated as

$$S_{WD,t} = \frac{\sum\{v|v \in T, t \neq v\} w_{t,v} \cdot S_{M,t,v} \cdot |y_t - y_v|}{\sum\{v|v \in T, t \neq v\} w_{t,v,e}} \quad (4)$$

where the subscript t and v represent two training compounds; the weight function for the pairwise compound t and v ($w_{t,v,e}$) was calculated with either eq (2).; yt and$y$ v stand for observed labels

Test S3. The optimal molecular fingerprint feature indices obtained for the Catboost model after SHAP-RFE

389, 650, 1602, 80, 1057, 378, 1457, 1917, 807, 1607, 745, 1683, 1171, 715, 926, 1873, 1452, 753, 1963, 1750, 1928, 1722, 1114, 1380, 790, 981, 1028, 1866, 1384, 1195, 275, 1088, 456, 728, 191, 283, 561, 1911, 1758, 319, 1385, 1754, 716, 1416, 366, 888, 1823, 202, 881, 1631, 392, 833, 875, 1,910, 1096, 586, 145, 932, 1097, 838, 1951, 1737, 1535, 1855, 695, 1430, 1991, 79, 786, 656, 694, 1498, 1480, 2004, 1724, 1588, 1339, 1172, 1570, 1357.
